# Operando surface science methodology reveals surface effect in charge storage electrodes

**DOI:** 10.1093/nsr/nwaa289

**Published:** 2020-12-08

**Authors:** Chao Wang, Yanxiao Ning, Haibo Huang, Shiwen Li, Chuanhai Xiao, Qi Chen, Li Peng, Shuainan Guo, Yifan Li, Conghui Liu, Zhong-Shuai Wu, Xianfeng Li, Liwei Chen, Chao Gao, Chuan Wu, Qiang Fu

**Affiliations:** State Key Laboratory of Catalysis, iChEM, Dalian Institute of Chemical Physics, Chinese Academy of Sciences, Dalian 116023, China; University of Chinese Academy of Sciences, Beijing 100049, China; State Key Laboratory of Catalysis, iChEM, Dalian Institute of Chemical Physics, Chinese Academy of Sciences, Dalian 116023, China; Dalian National Laboratory for Clean Energy, Dalian Institute of Chemical Physics, Chinese Academy of Sciences, Dalian 116023, China; State Key Laboratory of Catalysis, iChEM, Dalian Institute of Chemical Physics, Chinese Academy of Sciences, Dalian 116023, China; University of Chinese Academy of Sciences, Beijing 100049, China; State Key Laboratory of Catalysis, iChEM, Dalian Institute of Chemical Physics, Chinese Academy of Sciences, Dalian 116023, China; Suzhou Institute of Nano-Tech and Nano-Bionics, Chinese Academy of Sciences, Suzhou 215123, China; MOE Key Laboratory of Macromolecular Synthesis and Functionalization, Department of Polymer Science and Engineering, Zhejiang University, Hangzhou 310027, China; Beijing Key Laboratory of Environmental Science and Engineering, School of Materials Science & Engineering, Beijing Institute of Technology, Beijing 100081, China; State Key Laboratory of Catalysis, iChEM, Dalian Institute of Chemical Physics, Chinese Academy of Sciences, Dalian 116023, China; State Key Laboratory of Catalysis, iChEM, Dalian Institute of Chemical Physics, Chinese Academy of Sciences, Dalian 116023, China; Dalian National Laboratory for Clean Energy, Dalian Institute of Chemical Physics, Chinese Academy of Sciences, Dalian 116023, China; Dalian National Laboratory for Clean Energy, Dalian Institute of Chemical Physics, Chinese Academy of Sciences, Dalian 116023, China; Suzhou Institute of Nano-Tech and Nano-Bionics, Chinese Academy of Sciences, Suzhou 215123, China; MOE Key Laboratory of Macromolecular Synthesis and Functionalization, Department of Polymer Science and Engineering, Zhejiang University, Hangzhou 310027, China; Beijing Key Laboratory of Environmental Science and Engineering, School of Materials Science & Engineering, Beijing Institute of Technology, Beijing 100081, China; State Key Laboratory of Catalysis, iChEM, Dalian Institute of Chemical Physics, Chinese Academy of Sciences, Dalian 116023, China; Dalian National Laboratory for Clean Energy, Dalian Institute of Chemical Physics, Chinese Academy of Sciences, Dalian 116023, China

**Keywords:** surface science methodology, aluminum ion battery, operando characterization, surface effect

## Abstract

Surface and interface play critical roles in energy storage devices, calling for operando characterization techniques to probe the electrified surfaces/interfaces. In this work, surface science methodology, including electron spectroscopy and scanning probe microscopy, has been successfully applied to visualize electrochemical processes at operating electrode surfaces in an Al/graphite model battery. Intercalation of anions together with cations is directly observed in the surface region of a graphite electrode with tens of nanometers thickness, the concentration of which is one order higher than that in bulk. An intercalation pseudocapacitance mechanism and a double specific capacity in the electrode surface region are expected based on the super-dense intercalants and anion/cation co-intercalation, which are in sharp contrast to the battery-like mechanism in the electrode bulk. The distinct electrochemical mechanism at the electrode surface is verified by performance tests of real battery devices, showing that a surface-dominant, nanometer-thick graphite cathode outperforms a bulk-dominant, micrometer-thick graphite cathode. Our findings highlight the important surface effect of working electrodes in charge storage systems.

## Teaser

Surface science methodology has been successfully applied to visualize electrochemical processes at operating electrode surfaces, enabling to reveal an important surface effect of electrodes in charge storage systems

## INTRODUCTION

Fundamental understanding of elementary electrochemical processes at electrified surfaces/interfaces of electrochemical energy storage devices strongly relies on development and application of *insitu*/operando characterization techniques. Significant progresses have been made in recent decades [1,2], and successful characterization techniques include X-ray diffraction (XRD) [3–5], transmission electron microscopy (TEM) [6–9], X-ray spectroscopy and topography [4,10,11], nuclear magnetic resonance (NMR) [12], etc. Rich electronic, chemical, and geometric information from the bulk regions of electrodes and electrolytes can be derived while overlooking surface and interface processes. It is well known that device performance is mainly governed by surface and interface electrochemical reactions in most state-of-the-art nanosized electrodes [10,13,14], which thus call for operando surface and interface analysis.

Surface science methodology such as electron spectroscopy and scanning probe microscopy, has proven to be successful in providing a detailed description of how chemical reactions take place on solid surfaces [15]. Applications of the sophisticated surface science techniques to electrochemical devices should address key issues at the electrified surface/interface, which remain less explored, and more challenging in contrast with surface catalysis research [15–18]. First, surface science analysis is mostly done in ultrahigh vacuum (UHV), and thus it is difficult to explore electrochemical reactions occurring at liquid/solid interfaces. Nevertheless, solid-state or ionic liquid (IL) electrolytes are UHV compatible [6,7,9,10,19,20], and even water/solid interfaces can be probed by newly developed near ambient pressure surface techniques [18,21,22]. Second, most surface-sensitive techniques need to probe open and well-defined surfaces, but electrode surfaces in real electrochemical devices are all buried by electrolytes and current collectors which are totally inaccessible to surface probes. Thus, it is crucial to construct model electrochemical devices having planar and open electrified surfaces for surface analysis [9,10,19,23].

In this work, taking an aluminum (Al) ion battery (AIB) as an example [24,25], we built a planar Al/graphite model battery consisting of Al foil anode | 1-ethyl-3-methylimidazolium chloride (EMImCl)/AlCl_3_ (1 : 1.3 by mole ratio) IL electrolyte | highly ordered pyrolytic graphite (HOPG) cathode. Using the UHV compatible IL electrolyte and open electrode, the model devices were successfully placed into various surface systems including Raman, X-ray photoelectron spectroscopy (XPS) and atomic force microscopy (AFM) for multiple operando characterizations of the working electrode surfaces. The comprehensive surface analysis provides an unprecedented chance to follow elementary electrochemical reaction steps at electrode surfaces. More interestingly, a distinct electrochemical reaction mechanism was identified in the surface regions of a bulk electrode, which allowed us to predict successfully doubling of the specific capacity using surface-dominant, nanometer-thick graphite electrode materials in real coin-type batteries.

## RESULTS AND DISCUSSION

### Model AIB devices and their electrochemical behaviors

**Figure 1.**
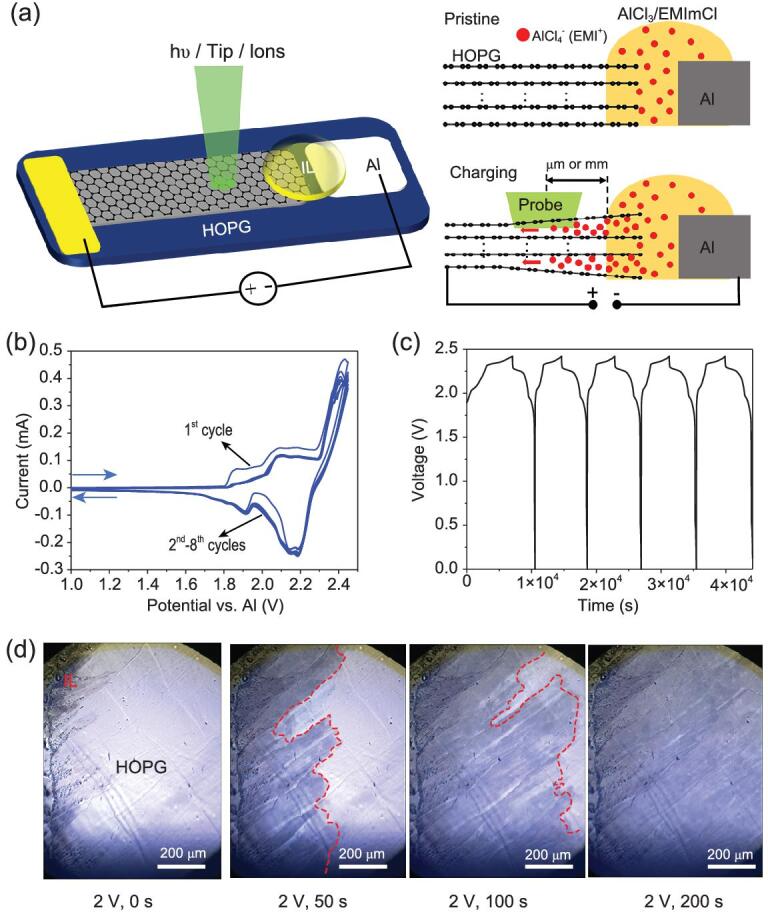
Model AIB devices and their electrochemical behaviors. (a) Schematic for Al/graphite model battery enabling simultaneous electrochemical measurements and surface science measurements using photons or scanning probes over the open electrodes (left panel). Side views of the model battery showing that ions intercalate into graphene layers from liquid/graphite interfaces and diffuse over μm or even mm distances (right panel). (b) Cyclic voltammetry (CV) curves (0–2.45 V, 0.5 mV/s) and (c) galvanostatic charge-discharge (GCD) curves (60 μA, cutoff voltage: 2.42 V, discharge plateaus: ∼2.2 V) from the model battery. (d) Operando OM images (700 μm × 950 μm) captured at the indicated charging times on 2 V over HOPG electrode. Diffusion frontiers are highlighted by red dashed lines.

For the operando surface science analysis, a planar model AIB with open electrode surface was designed. As illustrated in Fig. 1a (left), a freshly exfoliated HOPG flake (a few millimeters long and wide, and tens of micrometers thick) was employed as the working electrode (WE), which was placed parallel with an aluminum foil (counter electrode, CE). IL electrolyte was dropped on the gap between the electrodes. Notably, most of the HOPG surface was free from electrolyte and open to the surface probe. Alternatively, HOPG flake, glass fiber separator filled with IL electrolyte, and Al foil can be stacked to form a sandwich-like AIB model device (Fig. S1a). Electrochemical tests were performed over the model devices. As shown in Fig. 1b and Fig. S1b, intercalation peaks around ∼1.9, 2.1, and 2.4 V were observed in cyclic voltammetry (CV) curves upon charging the model devices. Furthermore, galvanostatic charge-discharge (GCD) curves of the model batteries are shown in Fig. 1c, and two charged plateaus in the range of 1.9–2.3 V and 2.3–2.4 V are clearly seen. Overall, the model devices displayed the same electrochemical behaviors as real Al/graphite batteries [24].

In addition to the electrochemical performance, diffusion of ions inside the HOPG electrode was recorded in real-time by optical microscope (OM) using an operando OM/Raman cell equipped with a transparent quartz window (Fig. S2). Figure 1d displays a set of images captured at 2 V for different charging times. It can be seen that dark contrast of the graphite surface is induced by the ion intercalation and the diffusion frontiers can be clearly distinguished by OM. Accordingly, ultrafast (∼3.7 μm/s) and ultralong (up to centimeters) diffusion of the intercalated ions underneath the HOPG surface was directly confirmed through the operando OM measurements. The varied optical contrasts suggest different charging states (Fig. S3, Videos 1–3). Based on open HOPG electrode and long-distance lateral ion transport within the electrode, our planar model battery with the HOPG WE provides an ideal model system for the operando surface science studies (Fig. 1a, right).

### Operando surface science analysis of the graphite electrode

**Figure 2.**
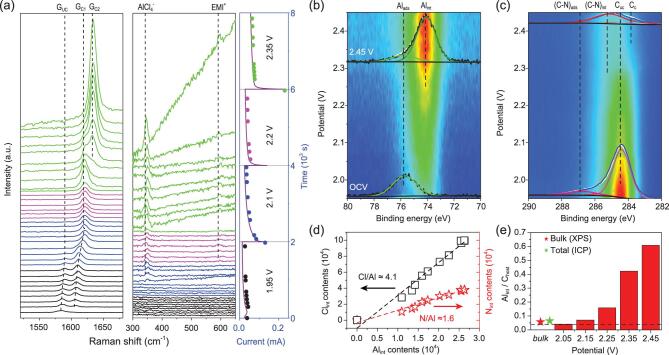
Operando surface science analysis of the graphite electrode. (a) Operando Raman spectra showing evolution of graphite G band (left) and intercalation ion signals (middle) upon charging at different potentials and different times (right). (b and c) A set of operando XPS Al 2p (b) and C 1s (c) spectra when charging from 1.95 to 2.45 V. The bottom Al 2p spectrum in (b) is from surface-adsorbed${\rm{\ AlC}}{{\rm{l}}_4}^ - $ species (Al_ads_) contaminated on the pristine electrode at open circuit voltage (OCV), while the top spectrum in (b) is mainly from intercalated ${\rm{AlC}}{{\rm{l}}_4}^ - \ $species (Al_int_) on the completely charged electrode (2.45 V for 2 h). The bottom and top C 1s spectra in (c) are from the electrode surface before and after the charging process, respectively. (d) Dependence of intercalated Cl (Cl_int_) and intercalated N (N_int_) contents with intercalated Al (Al_int_) contents in the charging process, showing that Cl/Al and N/Al atomic ratios are 4.1 and 1.6 at all potentials. (e) Atomic ratios between intercalated Al (Al_int_) and host-C (C_host_) at different potentials. Al/C_host_ ratios at the fully charged state from the theory limit (dashed line) [5], XPS measurement over the bulk electrode (red star), and chemical analysis of the whole graphite electrode (green star) are included (Fig. S16).

Structural changes of the graphite electrode were studied by operando Raman spectroscopy at the open electrode surface of the planar AIB device (Fig. 2a). Upon charging, the characteristic G band of pristine graphite (denoted as G_uc_, uncharged state) shifts to higher wavenumber positions, which can be assigned to graphene layers charged by adjacent ions (G_c_) [26]. Charging at 2.2 V produces a stage-2 graphite intercalation compound (GIC), as indicated by the dominant G_c1_ peak at ∼1620 cm^−1^. With the potential set at 2.35 V, a new peak around 1635 cm^−1^ (denoted as G_c2_) appears and finally becomes dominant, which can be attributed to more strongly charged graphene layers in stage-1 GIC [27–30]. Besides the graphite signals, intercalated electrolyte species were also detected by

Raman from the open electrode surface. When the potential is higher than 2.1 V, two peaks at ∼350 and ∼600 cm^−1^ start to appear (Fig. 2a), which are characteristic of ${\rm{AlC}}{{\rm{l}}_4}^ - $ and EMI^+^ ions, respectively [24]. Therefore, operando Raman measurements give direct evidence of co-intercalation of ${\rm{AlC}}{{\rm{l}}_4}^ - $ and EMI^+^ ions and formation of stage-1 GIC in the surface region of HOPG which depth is < 100 nm because of strong absorption of light by graphite [31]. Upon discharging, the evolution of G band is fully reversible, i.e. G_c2_ → G_c1_ + G_c2_ → G_c1_ → G_uc_ + G_c1_ → G_uc_, when probing the HOPG surface close to the electrolyte/graphite interface (Fig. S4).

The sandwich-like AIB devices (Fig. S1) were loaded into a system for operando XPS measurements. Chemical component and charge transfer in the surface region of the graphite electrode were investigated in detail. During the XPS measurements, graphite electrodes were always grounded and external potentials ramped from 1.95 to 2.45 V (V_graphite_ − V_Al_) were applied to Al anodes (Fig. S1). During the charging process, Al 2p, Cl 2p, and N 1s core-level signals appear at lower binding energy (BE) positions and show remarkable intensity increase (Fig. 2b and details in Figs S5–S7). These newly appeared spectra all present −1.7 eV BE shifts compared with those from the supported IL overlayers (Fig. S8 and Table S1). In contrast, the C 1s signal intensity decreases largely, as a result of attenuation by the intercalated ions (Fig. 2c and Fig. S9). A HOPG electrode was deliberately coated with an ultrathin gold layer and Au 4f peak position acquired from this operating electrode remained constant during the charging process (Fig. S10). This reference experiment confirms that the newly appeared Al 2p, Cl 2p, and N 1s signals are caused by electrochemical intercalation of electrolyte ions into HOPG. Work function of the operating HOPG electrode surface was measured by operando scanning Kelvin probe microscopy (SKPM), which increases by ∼1.7 eV in the fully charged state (Fig. S11). Thus, it is the downshift of the Fermi level at the charged graphite surface that results in the observed rigid BE shifts for all intercalated Al, Cl, N, and C elements [32] (Fig. S12).

The chemical composition of the intercalants and GICs can be further determined from the XPS data (Fig. 2d and e). The intercalation of ${\rm{AlC}}{{\rm{l}}_4}^ - $ together with EMI^+^ is unambiguously confirmed by the facts of Al_int_/Cl_int_ = 1 : 4.1 and Al_int_/N_int_ = 1 : 1.6, which agree with the above operando Raman results. By subtracting the contributions from guest C atoms in intercalated EMI^+^ (C-N at 285.2 eV/(C-N)_int_ and C-C at 283.6 eV/(C-C)_int_) [20,33], the atomic ratios between Al_int_ and C_host_ atoms (C_host_ including uncharged graphite at 284.5 eV/C_uc_ and charged graphite at 283.9 eV/C_c_, Fig. 2c and Fig. S13) at different potentials were obtained (Fig. 2e). We found that the Al_int_/C_host_ ratio increased to 1 : 1.7 at the fully charged state, which is about one order higher than the theoretical limit in AIB (1 : 24) [5]. For the first time, the electrochemical reaction of AIB in the surface region can be described by the following formula:

(1) \begin{eqnarray*}{{\rm C}_n} &+& 5[{\rm AlC}{{\rm l}_{4}}^{-} ] + 4[{\rm E\!M}{{\rm I}^{+} }]\rightarrow{{\rm C}_{n}}{[{\rm AlC}{{\rm l}_{4}}]_{5}}\nonumber\\&&\times{[{\rm E\!MI}]_{4}} + {e^{-} }(n \sim 8.5).\end{eqnarray*}

The small *n* (8.5) indicates an abnormal super-dense intercalant state, forming multi-layered cations/anions within the graphene layers like the formation of the super-dense lithium phase in bilayer graphene [9,34].

Such super-dense intercalation structure and large amount of cation co-intercalation may be unstable, and the electronic structure evolution induced by redistribution of the intercalated cations and anions under open circuit (OC) condition was investigated by operando XPS. The Al, Cl and N core levels shifted by +0.4 eV at the OC state for 72 h (Fig. S14). Such relaxation may be attributed to charge redistribution in the cation/anion multi-layers in between the graphite layers, similar to that in supercapacitors [35], which does not happen in the bulk regions (Fig. S15).

### Distinct electrochemical process at electrode surface compared to bulk

**Figure 3.**
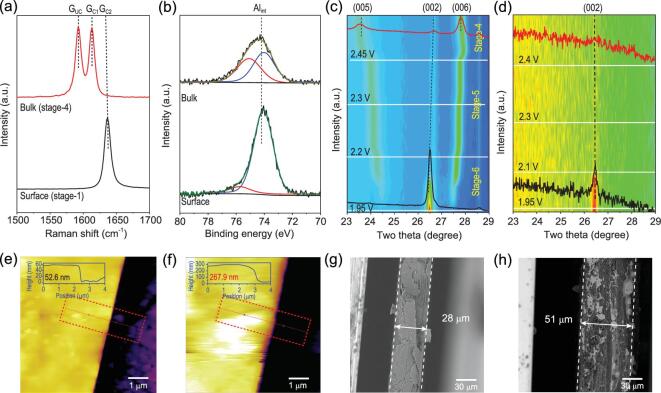
Depth/thickness-dependent electrochemical intercalation process. Distinct electrochemical intercalation process at surface region. (a and b) Raman spectra (a) and XPS Al 2p spectra (b) acquired from as-charged graphite surface and bulk region of the electrode after exfoliation of the surface layer, respectively. (c and d) Operando XRD patterns from (c) thick graphite film (*d* = ∼20 μm) electrode and (d) thin graphite film (*d* = ∼50 nm) electrode. The bottom and top XRD patterns in (c) and (d) are from the electrode before and after the charging process, respectively. (e and f) *In situ* atomic force microscope (AFM) images of a nano graphite flake at OCV (e) and fully charged state (f), respectively. The inset line profiles show the step heights labeled by the red rectangles. (g and h) *Ex situ* SEM cross-section images of a graphite flake at OCV (g) and fully charged state (h), respectively.

To make a comparison of the electrochemistry at the electrode surface and in bulk, quasi *insitu* Raman and XPS measurements were carried out on a fully charged electrode subjected to mechanical exfoliation (exposure of the *bulk* region to surface analysis) (Supplementary data, Scheme 1). Figure 3a shows depth-dependent graphite intercalation structures: stage-1 GIC at the surface *vs.* stage-4 GIC in the bulk. Our experimental evidence for different intercalation stages between the electrode surface and bulk confirms the theoretical hypothesis of core-shell structure of intercalated electrode particles [30]. XPS Al 2p, N 1s, and C 1s (C-N)_int_ peaks from the bulk region have similar BE positions to those from the as-charged surface but their intensities are all strongly weakened (Fig. 3b and Fig. S16), while the C 1s signal from C_host_ atoms becomes much stronger (Fig. S16). The determined Al_int_/C_host_ ratio is 1 : 19.1 and Al_int_/N_int_ ratio is 1 : 0.7 (Fig. 2e and Fig. S16). Both intercalated$\ {\rm{AlC}}{{\rm{l}}_4}^ - \ $and co-intercalated EMI^+^ concentrations in bulk are much smaller than those in the surface region. To validate the semi-quantitative results from the XPS analysis, the chemical composition of the fully charged graphite electrode was analyzed by chemical analysis methods, producing similar results. Figure 2e and Fig. S16 indicate Al_int_/C_host_ = 1 : 17.5 and Al_int_/N_int_ = 1 : 1.1, which are considered to be normal in comparison with previous reports [5,25]. The depth-dependent intercalation reaction can be also confirmed by *exsitu* time-of-flight secondary ion mass spectrometry (TOF-SIMS) measurements. The mass spectrometry (MS) signals related to the intercalation concentration (Al^−^/C_3_^−^) keep on decreasing with the increasing sputtering time (Fig. S17), revealing that the intercalant concentration gradient is present in the surface region. The thickness (*d*) of the unusual surface region is estimated to be within 100 nm (Fig. S18).

The distinct electrochemical behavior in the surface region is further verified by operando XRD measurements (Fig. S19) over two kinds of graphite materials: nanometer-thick graphite films (*d* = ∼50 nm) *vs.* micrometer-thick graphite films (*d* = ∼20 μm). An ordered stage-4 GIC structure forms in the thick graphite film electrode (Fig. 3c), while the fully charged ultrathin graphite film electrode has a disordered structure (Fig. 3d). *Exsitu* grazing incidence X-ray diffraction (GIXRD) measurement probes the surface region directly and confirms the same surface structure (Fig. S20). The volume expansion of ultrathin electrode during intercalation was measured by *insitu* AFM analysis over a 52 nm-thick graphite flake, which reveals more than 5-fold volume expansion (Fig. 3e and f, Figs S21 and S22). The micrometer-thick graphite flake shows the normal 2-fold thickness increase by imaging its cross-section with *exsitu* scanning electron microscopy (Fig. 3g and h). The much larger volume expansion and less-ordered phase in the nanometer-thick cathode is consistent with the above finding of super-dense intercalated multi-layered ions in graphite layers at the surface region as concluded from the operando XPS measurements (Fig. 2e).

### Electrode surface effect on battery performance

**Figure 4.**
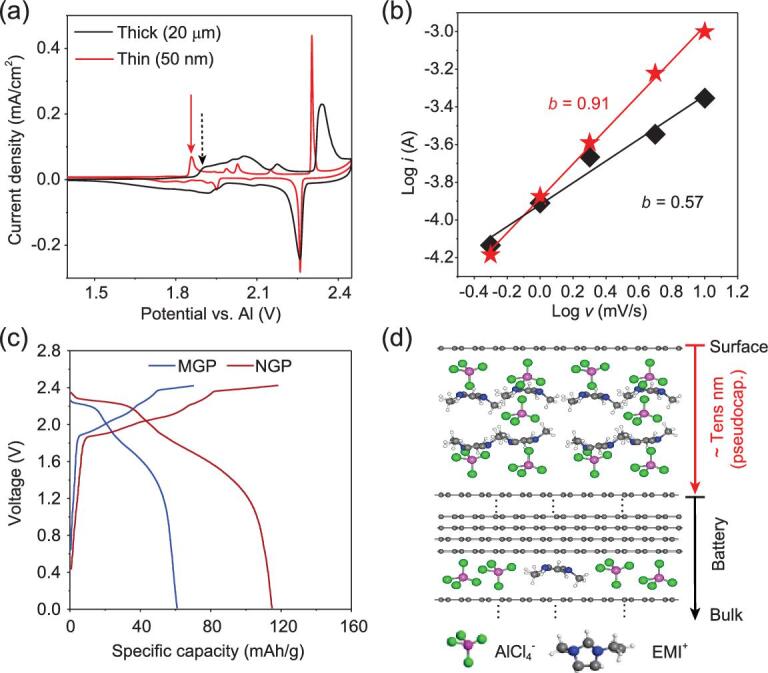
Surface/thickness effect on battery performance. (a) CV curves of the electrochemical devices based on thick graphite film (*d* = ∼20 μm) and ultrathin graphite film (*d* = ∼50 nm) electrodes (scan rate: 0.5 mV/s) in the three-electrode mode. (b) Dependence of peak current (*i*) on the scan rate (*v*) and the derived slopes (*b*) of Log(*i*)/Log(*v*) lines for the 1st intercalation peak around 1.9 V, marked by arrows in (a). (c) GCD curves of real AIB devices after the 20th cycle at a current density of 0.5 A/g using nano graphite powder (NGP) and micrometer graphite powder (MGP) as cathode materials (cutoff voltage: 2.42 V). (d) Schematic illustration of depth-dependent charge storage processes: multi-layer ${\rm{AlC}}{{\rm{l}}_4}^ - $ and EMI^+^ intercalated in near surface region with intercalation pseudocapacitance mechanism *vs.* dominant ${\rm{AlC}}{{\rm{l}}_4}^ - $ intercalation in bulk region with battery process.

The above characterization results demonstrate a remarkable surface effect on the electrochemical reaction in AIB, which may subsequently affect the device performance. We first explored the intercalation kinetics in electrodes of nanometer-thick graphite film (*d* = ∼50 nm, area of 1 × 1 cm^2^) and micrometer-thick graphite film (*d* = ∼20 μm, area of 1 × 1 cm^2^), in which surface region and bulk region are dominant in the two electrodes, respectively. CV tests are performed at different scan rates in the three-electrode mode. According to Fick's law of semi-infinite diffusion, peak current (*i*) and scan rate (*v*) follow the formula *i = a ⋅ v^b^* [36]*.* Much sharper redox peaks at lower intercalation potential were observed in the CV curves acquired from the device using the ultrathin graphite film cathode (Fig. 4a and Fig. S23) with a derived *b* value of ∼0.91 (Fig. 4b), revealing a charge-transfer-limited intercalation pseudocapacitance process [37]. In contrast, the *b* value measured from the device using the thick graphite film cathode is 0.57, indicating a diffusion-limited battery-like process. The two contrast charging mechanisms are also present in the real coin-type devices (Fig. S24). The sharper CV peaks observed from the nanometer-thick graphite electrodes also reveal the higher diffusion rate of intercalated ions within the ultrathin electrodes and the formed uniform local structure around the intercalated ions [14].

The above characterization data confirm that electrochemical processes within electrode surface regions or in ultrathin electrodes are dominated by the intercalation pseudocapacitance charging mechanism as manifested by one order higher ${\rm{AlC}}{{\rm{l}}_4}^ - $concentration compared with the theoretical value but co-intercalation of [${\rm{AlC}}{{\rm{l}}_4}^ - $]_5_ and [EMI^+^]_4_. Accordingly, the theoretical capacity is expected to be doubled in this case. Guided by this insight, we assembled two coin-type devices using nano graphite powder (NGP) (3–10 nm thick and 5–10 μm large) and micrometer graphite powder (MGP) (diameter ∼15 μm) as cathode materials, respectively. As shown in Fig. 4c, the capacity has been improved from 61 mAh · g^−1^ with the MGP cathode to 116 mAh·g^−1^ with the NGP cathode at 0.5 A · g^−1^, and such performances are maintained for 100 cycles (Fig. S25a) and at higher current density (Fig. S25b). Notably, the diffusion lengths in both NGP and MGP cathodes are in the same scale of micrometers (Fig. S26), and the only difference is the thickness. As shown above (Fig. 1d), intercalation of electrolyte ions and their lateral diffusion in graphite cathodes are feasible and ultrafast in the Al/graphite battery [24,25]. Therefore, the observed different electrochemical reaction mechanisms and device performances between the surface and bulk regions should not be attributed to the diffusion limit or surface adsorption, which are commonly used to explain size effect in energy storage processes [14,38,39]. It is suggested that the demonstrated surface effect may originate from the enhanced structural flexibility of the electrode surface regions [40], allowing accommodation of more intercalation ions in graphene layers (Fig. 4d), and this explains the origin of better performance with the nanometer-thick graphene electrodes compared with the micrometer-thick graphite electrodes [25,41].

## CONCLUSION

Distinct electrochemical intercalation reactions in electrode surface regions were revealed by comprehensive operando surface science measurements over well-designed Al/graphite model batteries. Multi-layer super-dense anions with co-intercalated cations, with concentration about one order higher than that in electrode bulk, were observed. The depth-dependent charge storage mechanism can be described as the intercalation pseudocapacitance mechanism dominant in the surface regions in contrast with the battery-like process occurring in the bulk regions. The revealed surface effect on electrochemical storage guides to double the capacity using a nanometer-thick graphite electrode. This work suggests a new strategy for operando studies of surface electrochemical reaction over electrode surface using surface-sensitive techniques, and highlights the significance of electrode surfaces in electrochemical device performance.

## METHODS

### Operando Raman and XPS measurements

Operando Raman characterizations were based on the planar model batteries. The model batteries were placed into an *insitu* Raman cell (Fig. S2). The battery devices and then the cell were assembled in an Ar-filled glove box (H_2_O, O_2_ < 0.5 ppm). Raman spectra were recorded with a LabRAM HR 800 Raman spectrometer using a 532 nm laser. The incident laser was illuminating on the open area of the HOPG flake.

The model battery used for XPS measurements has a sandwich structure (Fig. S1), which was assembled onto Omicron-type direct-current heating sample holders in a glove box and then transferred to the XPS analysis system using a mobile UHV transfer chamber. The HOPG flake (5 × 12 mm^2^) was connected with the sample holder and thus grounded. A small piece of glass fiber separator layer adsorbed with IL was placed at one end of the HOPG flake. Subsequently, a small piece of Al foil was put on top of the separator and then connected to a contact bar, which was insulated from the sample holder.

XPS core level spectra were recorded under UHV conditions (*P* < 10^−8^ mbar) using pass energy of 20 eV. Data analysis was done by Casa-XPS software with a Shirley background and 70/30 Gaussian-Lorentzian fits. The chemical composition (Fig. 2d and e) of the GICs was determined by XPS fitting results (as shown in Fig. S13). Al_int_/C_host_ was calculated with the formula: Al_int_/C_host _= Al_int_/(C_total_−N_int _× 3) according to the stoichiometric ratio of EMI^+^.

Comparisons between surface and bulk were measured by quasi *insitu* XPS and Raman measurements on exfoliated as-charged HOPG electrodes **(**Supplementary data, Scheme 1). For each process including assembling, transfer, charging and exfoliation, exposure to air was avoided in both operando and quasi *in situ* measurements.

### *In* *situ* AFM measurements

*In* *situ* AFM was performed over a nano graphite flake (mechanical exfoliation from a HOPG crystal) on a flat glassy carbon (GC) substrate (Gaoss Union company Wuhan, China) operated by a Cypher ES AFM (Asylum Research, Oxford Instruments, USA) installed in an Ar-filled glove box (Fig. S21). Before testing, the sample is calcined under Ar at 900°C for 3 h to remove the residue tape. The AFM tip (AC160TSA-R3-10, 250 Hz, 20 N/m) is totally immerged in the electrolyte drop and can measure the nanosheet height during the charging. For comparison, the expansion of thick graphite flake was measured by *exsitu* SEM (Phenom ProX, Phenom World, Netherlands) imaging of the cross-section over the same sample edge.

### Operando XRD measurements

Large area high-quality graphite films of different thicknesses (Fig. S23) were synthesized by high temperature thermo-reduction of graphene oxide (GO) layers [25,42]. Operando XRD measurements of the graphite electrodes were studied with a Rigaku Ultima IV X-ray diffractometer (Cu Kα, 40 kV, 40 mA) in an *insitu* XRD cell (Fig. S19). The electrochemical operando XRD patterns were acquired in 2θ range of 15°–35° with a scan rate of 5° · min^−1^. Operando XRD analysis was performed using constant charging potential as illustrated in Fig. 3c and d. The stage number n of the GIC is calculated by the ratio between two interplanar spacings of dominant diffraction peaks: (0, 0, n + 1) and (0, 0, n + 2) [43]. *Exsitu* GIXRD measurements were performed by SmartLab XRD (Rigaku, Japan). The angle of incidence X-ray is 1° and 0.3° (Fig. S20). The *ex situ* GIXRD patterns were acquired in 2θ range of 20°–30°, with a scan rate of 5° · min^−1^.

## Supplementary Material

nwaa289_Supplemental_File

## References

[1] Grey CP , Tarascon JM. Sustainability and in situ monitoring in battery development. Nat Mater 2016; 16: 45–56. 10.1038/nmat4777 27994251

[2] Boebinger MG , Lewis JA, Sandoval SE et al. Understanding transformations in battery materials using in situ and operando experiments: progress and outlook. ACS Energy Lett 2020; 5: 335–45. 10.1021/acsenergylett.9b02514

[3] Kirshenbaum K , Bock DC, Lee CY et al. In situ visualization of Li/Ag_2_VP_2_O_8_ batteries revealing rate-dependent discharge mechanism. Science 2015; 347: 149–54. 10.1126/science.1257289 25574017

[4] Lee J , Kitchaev DA, Kwon DH et al. Reversible Mn^2+^/Mn^4+^ double redox in lithium-excess cathode materials. Nature 2018; 556: 185–90. 10.1038/s41586-018-0015-4 29643482

[5] Pan CJ , Yuan CZ, Zhu GZ et al. An operando X-ray diffraction study of chloroaluminate anion-graphite intercalation in aluminum batteries. Proc Natl Acad Sci USA 2018; 115: 5670–5. 10.1073/pnas.1803576115 29760096 PMC5984537

[6] Huang JY , Zhong L, Wang CM et al. In situ observation of the electrochemical lithiation of a single SnO_2_ nanowire electrode. Science 2010; 330: 1515–20. 10.1126/science.1195628 21148385

[7] Zhang W , Seo DH, Chen T et al. Kinetic pathways of ionic transport in fast-charging lithium titanate. Science 2020; 367: 1030–4. 10.1126/science.aax3520 32108110

[8] Li YZ , Li YB, Pei AL et al. Atomic structure of sensitive battery materials and interfaces revealed by cryo-electron microscopy. Science 2017; 358: 506–10. 10.1126/science.aam6014 29074771

[9] Kuhne M , Borrnert F, Fecher S et al. Reversible superdense ordering of lithium between two graphene sheets. Nature 2018; 564: 234–9. 10.1038/s41586-018-0754-2 30478294

[10] Liu XS , Wang DD, Liu G et al. Distinct charge dynamics in battery electrodes revealed by in situ and operando soft X-ray spectroscopy. Nat Commun 2013; 4: 3568. 10.1038/ncomms3568 PMC3806410 24100759

[11] Ebner M , Marone F, Stampanoni M et al. Visualization and quantification of electrochemical and mechanical degradation in Li ion batteries. Science 2013; 342: 716–20. 10.1126/science.1241882 24136360

[12] Zhao EW , Liu T, Jonsson E et al. In situ NMR metrology reveals reaction mechanisms in redox flow batteries. Nature 2020; 579: 224–8. 10.1038/s41586-020-2081-7 32123353

[13] Lim J , Li YY, Alsem DH et al. Origin and hysteresis of lithium compositional spatiodynamics within battery primary particles. Science 2016; 353: 566–71. 10.1126/science.aaf4914 27493180

[14] Jung S-K , Hwang I, Chang D et al. Nanoscale phenomena in lithium-ion batteries. Chem Rev 2020; 120: 6684–737. 10.1021/acs.chemrev.9b00405 31793294

[15] Ertl G. Reactions at surfaces: from atoms to complexity (Nobel lecture). Angew Chem Int Ed 2008; 47: 3524–35. 10.1002/anie.200800480 18357601

[16] Fu Q , Li WX, Yao YX et al. Interface-confined ferrous centers for catalytic oxidation. Science 2010; 328: 1141–4. 10.1126/science.1188267 20508127

[17] Jaramillo TF , Jorgensen KP, Bonde J et al. Identification of active edge sites for electrochemical H_2_ evolution from MoS_2_ nanocatalysts. Science 2007; 317: 100–2. 10.1126/science.1141483 17615351

[18] Tao F , Salmeron M. In situ studies of chemistry and structure of materials in reactive environments. Science 2011; 331: 171–4. 10.1126/science.1197461 21233377

[19] Zhang CJ , Grass ME, McDaniel AH et al. Measuring fundamental properties in operating solid oxide electrochemical cells by using in situ X-ray photoelectron spectroscopy. Nat Mater 2010; 9: 944–9. 10.1038/nmat2851 20871607

[20] Steinruck HP , Libuda J, Wasserscheid P et al. Surface science and model catalysis with ionic liquid-modified materials. Adv Mater 2011; 23: 2571–87. 10.1002/adma.201100211 21520462

[21] Favaro M , Jeong B, Ross PN et al. Unravelling the electrochemical double layer by direct probing of the solid/liquid interface. Nat Commun 2016; 7: 12695. 10.1038/ncomms12695 27576762 PMC5013669

[22] Ning Y , Fu Q, Li Y et al. A near ambient pressure photoemission electron microscope (NAP-PEEM). Ultramicroscopy 2019; 200: 105–10. 10.1016/j.ultramic.2019.02.028 30851711

[23] Yang AK , Zhou GM, Kong X et al. Electrochemical generation of liquid and solid sulfur on two-dimensional layered materials with distinct areal capacities. Nat Nanotechnol 2020; 15: 231–8. 10.1038/s41565-019-0624-6 31988508

[24] Lin MC , Gong M, Lu BG et al. An ultrafast rechargeable aluminium-ion battery. Nature 2015; 520: 325–8. 10.1038/nature14340 25849777

[25] Chen H , Xu HY, Wang SY et al. Ultrafast all-climate aluminum-graphene battery with quarter-million cycle life. Sci Adv 2017; 3: eaao7223. 10.1126/sciadv.aao7233 PMC5733111 29255803

[26] Chacon-Torres JC , Wirtz L, Pichler T. Manifestation of charged and strained graphene layers in the Raman response of graphite intercalation compounds. ACS Nano 2013; 7: 9249–59. 10.1021/nn403885k 24025089 PMC3807528

[27] Cao JY , He P, Mohammed MA et al. Two-step electrochemical intercalation and oxidation of graphite for the mass production of graphene oxide. J Am Chem Soc 2017; 139: 17446–56. 10.1021/jacs.7b08515 29090921

[28] Yang CY , Chen J, Ji X et al. Aqueous Li-ion battery enabled by halogen conversion-intercalation chemistry in graphite. Nature 2019; 569: 245–50. 10.1038/s41586-019-1175-6 31068723

[29] Childress AS , Parajuli P, Zhu JY et al. A Raman spectroscopic study of graphene cathodes in high-performance aluminum ion batteries. Nano Energy 2017; 39: 69–76. 10.1016/j.nanoen.2017.06.038

[30] Novko D , Zhang Q, Kaghazchi P. Nonadiabatic effects in Raman spectra of AlCl_4_^−^-graphite based batteries. Phys Rev Appl 2019; 12: 024016. 10.1103/PhysRevApplied.12.024016

[31] Inaba M , Yoshida H, Ogumi Z et al. In-situ Raman-study on electrochemical Li-intercalation into graphite. J Electrochem Soc 1995; 142: 20–6. 10.1149/1.2043869

[32] Schnyder B , Alliata D, Kotz R et al. Electrochemical intercalation of perchlorate ions in HOPG: an SFM/LFM and XPS study. Appl Surf Sci 2001; 173: 221–32. 10.1016/S0169-4332(00)00902-8

[33] Foelske-Schmitz A , Weingarth D, Kaiser H et al. Quasi in situ XPS study of anion intercalation into HOPG from the ionic liquid EMIMBF_4_. Electrochem Commun 2010; 12: 1453–6. 10.1016/j.elecom.2010.08.007

[34] Kuhne M , Paolucci F, Popovic J et al. Ultrafast lithium diffusion in bilayer graphene. Nat Nanotechnol 2017; 12: 895–900. 10.1038/nnano.2017.108 28581509

[35] Kaus M , Kowal J, Sauer DU. Modelling the effects of charge redistribution during self-discharge of supercapacitors. Electrochim Acta 2010; 55: 7516–23. 10.1016/j.electacta.2010.01.002

[36] Augustyn V , Come J, Lowe MA et al. High-rate electrochemical energy storage through Li^+^ intercalation pseudocapacitance. Nat Mater 2013; 12: 518–22. 10.1038/nmat3601 23584143

[37] Choi C , Ashby DS, Butts DM et al. Achieving high energy density and high power density with pseudocapacitive materials. Nat Rev Mater 2020; 5: 5–19. 10.1038/s41578-019-0142-z

[38] Zhu CB , Usiskin RE, Yu Y et al. The nanoscale circuitry of battery electrodes. Science 2017; 358: eaao2808. 10.1126/science.aao2808 29242319

[39] Arico AS , Bruce P, Scrosati B et al. Nanostructured materials for advanced energy conversion and storage devices. Nat Mater 2005; 4: 366–77. 10.1038/nmat1368 15867920

[40] Jung SC , Kang YJ, Yoo DJ et al. Flexible few-layered graphene for the ultrafast rechargeable aluminum-ion battery. J Phys Chem C 2016; 120: 13384–9. 10.1021/acs.jpcc.6b03657

[41] Huang HB , Zhou F, Lu PF et al. Design and construction of few-layer graphene cathode for ultrafast and high-capacity aluminum-ion batteries. Energy Storage Mater 2020; 27: 396–404. 10.1016/j.ensm.2020.02.011

[42] Peng L , Xu Z, Liu Z et al. Ultrahigh thermal conductive yet superflexible graphene films. Adv Mater 2017; 29: 1700589. 10.1002/adma.201700589 28498620

[43] Schmuelling G , Placke T, Kloepsch R et al. X-ray diffraction studies of the electrochemical intercalation of bis(trifluoromethanesulfonyl)imide anions into graphite for dual-ion cells. J Power Sources 2013; 239: 563–71. 10.1016/j.jpowsour.2013.03.064

