# Supplementary material for: Operando surface science methodology reveals surface effect in charge storage electrodes
Source: Natl Sci Rev. 2020 Dec 8;8(3):nwaa289. doi: 10.1093/nsr/nwaa289 (PMC8288451; doi:10.1093/nsr/nwaa289)
Supplement: nwaa289_Supplemental_File [file nwaa289_supplemental_file.zip › Supplementary_Material_Revised.docx]

Supplementary Material

**Operando Surface Science Methodology Reveals Surface Effect in Charge Storage Electrodes**

Chao Wang^1,2^, Yanxiao Ning^1^, Haibo Huang^3^, Shiwen Li^1,2^, Chuanhai Xiao^1^, Qi Chen^4^, Li Peng^5^, Shuainan Guo^6^, Yifan Li^1^, Conghui Liu^1^, Zhongshuai Wu^3^, Xianfeng Li^3^, Liwei Chen^4^, Chao Gao^5^, Chuan Wu^6^, Qiang Fu^1,3^†

^1^State Key Laboratory of Catalysis, iChEM, Dalian Institute of Chemical Physics, Chinese Academy of Sciences, Dalian 116023, China; ^2^University of Chinese Academy of Sciences, Beijing 100049, China; ^3^Dalian National Laboratory for Clean Energy, Dalian Institute of Chemical Physics, Chinese Academy of Sciences, Dalian 116023, China; ^4^Suzhou Institute of Nano-Tech and Nano-Bionics, Chinese Academy of Sciences, Suzhou 215123, China; ^5^MOE Key Laboratory of Macromolecular Synthesis and Functionalization, Department of Polymer Science and Engineering, Zhejiang University, Hangzhou 310027, China; ^6^Beijing Key Laboratory of Environmental Science and Engineering, School of Materials Science & Engineering, Beijing Institute of Technology, Beijing 100081, China.

*Corresponding author: Email: [qfu@dicp.ac.cn](mailto:qfu@dicp.ac.cn) (Q.F.)

**Supplementary methods**

**Construction of Al | IL | highly oriented pyrolytic graphite (HOPG) planar battery** A piece of HOPG (peeled from a HOPG crystal (Bruker, Grade-ZYB, 12 × 12 mm^2^) flake and Al foil (Alfa Asesar, 99.45%, 5 × 5 mm^2^, thickness of 0.025 mm) were parallelly placed on the same plate with ~1 mm gap in between. The IL electrolyte adsorbed in a glass fiber separator (Whatman GF/D, 5 mm × 5 mm) was placed in between. Notably, only small part of the HOPG flake was in contact with the electrolyte drop. The copper tape was employed as current collector which was not in touch with the electrolyte to avoid any side reactions.

All electrochemical tests over the model AIB batteries were performed by using a CHI 660E electrochemical work station (Chen Hua company, Shanghai, China).

**Preparation of room temperature ionic liquid (RTIL) electrolyte**

1-ethyl-3-methylimidazolium chloride (EMImCl, 99%, Lanzhou Institute of Chemical Physics, the Chinese Academy of Science) was heated in a clean quartz tube under vacuum at 120 ℃ for 20 h. For the thorough dehydration, the tube was connected with a turbo pump to keep the vacuum better than 5 × 10^-6^ mbar. After that, EMImCl was transferred into an Ar-filled glove box (MBRAUN, H_2_O & O_2_ < 0.5 ppm) for mixing with anhydrous aluminum chloride (AlCl_3_, Alfa Aesar, 99.999%). The mole ratio of EMImCl to AlCl_3_ is 1:1.3, which is regarded to be optimum for the battery performance [1]. All the solids were dissolved under continual magnetic stirring and finally formed a transparent light-yellow liquid. To further purify the electrolyte, a piece of aluminum foil (Al foil, Alfa Asesar, 99.45%) was added into the electrolyte and kept under vacuum (< 5 × 10^-6^ mbar) until there is no bubbling [2]. All our experiments including electrochemical performance tests and operando studies are based on the same concentration electrolyte.

**Detailed information of the operando Raman and XPS measurements**

Operando Raman characterizations were based on the above-mentioned planar model batteries. The model batteries were placed into an in-situ Raman cell, which has a CF35 flange with a quartz window and is sealed by the polytetrafluoroethylene gasket. Two copper wires are used to connect the work electrode (WE) and counter electrode (CE) contact pins. The copper wires are not in touch with the electrolyte. The battery devices and the cell were assembled in the Ar-filled glove box (H_2_O, O_2_ < 0.5 ppm). Raman spectra were recorded with a LabRAM HR 800 Raman spectrometer using a 532 nm laser. To avoid the interference from the electrolytes, the incident laser was illuminating on the open area of the HOPG flake and the laser spot can be controlled at different distances from the electrolyte/HOPG interface, which is changed from a few hundred micrometers to a few millimeters. Two regimes of Raman data were acquired in this work: 1500 ~ 1700 cm^-1^ (G band of graphite) and 250 ~ 650 cm^-1^ (including signals of AlCl_4_^-^, EMI^+^, and Al_2_Cl_7_^-^). All the spectra were calibrated by Si single crystal at 520.7 cm^-1^. The stage number (n > 2) in the graphite intercalation compound (GIC) is calculated by the following equation [2]:

$\frac{I_{G_{\mathrm{uc}}}}{I_{G_{c}}}=\frac{\sigma_{G_{\mathrm{uc}}}}{\sigma_{G_{c}}}$ $\frac{(n-2)}{2}$

where I_Guc_ and I_Gc_ denote the intensities of E_2g_ mode (G band) of the uncharged and charged graphene layers, respectively. The value σ_Guc_/σ_Gc_ is the ratio of the cross section for Raman scattering from the uncharged and charged layers (a stage-independent constant)

The model battery used for XPS measurements has been slightly modified. The HOPG flake was cut into 5 × 12 mm^2^, which was connected with the sample holder and thus grounded. A small piece of glass fiber separator layer adsorbed with IL was placed on the top of one end of the HOPG flake. Subsequently, a small piece of Al foil was put on the top of the separator and then connected with a contact bar (made by Al) which were all insulated from the sample holder. The model battery was assembled onto an Omicron-type direct-current heating sample holder for XPS analysis. The assembling of the model devices and their mounting onto the sample holder were all done in the Ar-filled glove box and then transferred from the glove box to the XPS analysis system by using a mobile UHV transfer chamber. During the assembling and transfer processes, exposure to air has been strictly avoided.

During operando XPS measurements, HOPG flakes are always grounded while external potentials are applied onto Al anodes which are controlled by the work station (CHI 660E). For the binding energy calibration, Au decoration was performed by coating a thin gold film (< 10 nm) on a HOPG flake with a Neocoater MP-19020 NCTR (JEOL, Akishima-Shi, Tokyo, Japan) before the battery assembling. XPS measurements were carried out using a SPECS XPS spectrometer equipped with Al Kα X-ray source (1486.6 eV at 300 W) and a PHOIBOS 100 hemispherical energy analyzer. Survey spectra and core level spectra were recorded under ultrahigh vacuum (UHV) conditions (p < 10^-8^ mbar) using pass energy of 20 eV. Data analysis was done by Casa-XPS software with a Shirley background and 70/30 Gaussian-Lorentzian fits. All the quantitative analysis is based on the fitting results and corrected by atomic sensitivity factors (ASF).

**Operando observation of intercalation of HOPG surface by optical microscope** Optical microscope (OM) images were acquired by a reflective optical microscope (Oxford instrument, USA) on the Al | IL | HOPG planar battery operated in the in-situ Raman cell (Supplementary Fig. 2).

**Operando SKPM measurements**

Operando SKPM analysis was done in a Cypher ES AFM (Asylum Research, Oxford Instruments, USA) installed in an Ar-filled glove box. The surface imaging was performed on the open area of the working HOPG electrode in the Al | IL | HOPG planar model battery. The resonance frequency ω­_0_ and spring constant of AFM conducting tips (HQ-NSC14/Cr-Au) are around 140 kHz and 5.0 N/m, respectively.

**Ex-situ TOF-SIMS measurements**

Ex-situ TOF-SIMS measurements were carried out by TOF SIMS^5^ (ion-TOF company, Germany). The sputtering energy and current of negative ions are 2 KeV and 39.5 nA (Cs), respectively. The sputtering energy and current of the positive ions are 2 KeV and 250 nA (O), respectively. Multilayer graphene (~500 nm) grown on Ni via CVD was purchased from XF Nano (Nanjing, China). The fully charged thin graphene film and HOPG flakes were measured by the TOF-SIMS.

**Chemical analysis of chemical composition of charged graphite electrodes** Chemical composition in the electrode material (shown in Fig. 2E and fig. S16) was measured by chemical analysis methods including inductively coupled plasma (ICP) and elemental analyzer. The mass of host graphite (C_host_) was directly measured from the clean HOPG flake which was to be used for assembly to the model battery. The fully charged HOPG flake was dissembled from the battery device (after the operando XPS analysis) and then washed with ethanol to remove the surface absorbed electrolyte. The HOPG flake was [ultrasonic](javascript:;)ally [dissolved](javascript:;) by [chloroazotic](javascript:;) [acid](javascript:;) and the Al concentration was measured by ICP (ICPS-8100, Shimadzu, Japan). Finally, the mass of intercalated Al atoms in the HOPG flake can be calculated. Accordingly, Al_int_/C_host_ atomic ratio can be determined.

The mass of C in the charged electrode was measured by the C/S analyzer (EMIA-8100, Horiba, Japan), and the masses of N, H, and O elements in the charged electrode were measured by the O/N/H analyzer (EMGA-930, Horiba, Japan).

**Electrochemical measurements of batteries based on graphite film and powder electrodes with different thickness**

CV tests at different scan rates were carried out in three-electrode mode: the graphite films with different thickness (area: 1 cm^2^) were employed as working electrode (WE) and directly contacted with the Ta current collector. The Al foils were employed as the counter and reference electrode (CE/RE). The WE and CE/RE were stacked as the sandwich structure and the glass fiber separator was placed in between.

Two kinds of graphitic materials including graphite powders (800 mesh, Hua Tai Machinery, Qingdao, China) with particle size of ~15 μm and graphene nanosheets with the lateral size of 5 - 10 μm and thickness of 3 - 10 nm (from XF Nano, China) were used as the cathodes. The mass ratio of the active graphitic material and binder (Polyvinylidene Fluoride, PVDF) was 9:1. They were mixed in N-methyl-2-pyrrolidone (NMP) and then pasted onto the surface of a Ta foil current collector. The coated electrode was dried at 100 °C for 24 h in vacuum. Al foil (thickness of 0.1 mm, Alfa Asesar, 99.99%) was used as the anode and glass fiber paper (Whatman 934-AH) as the separator. The coin-type cell fabrication was done in an Ar-filled glove


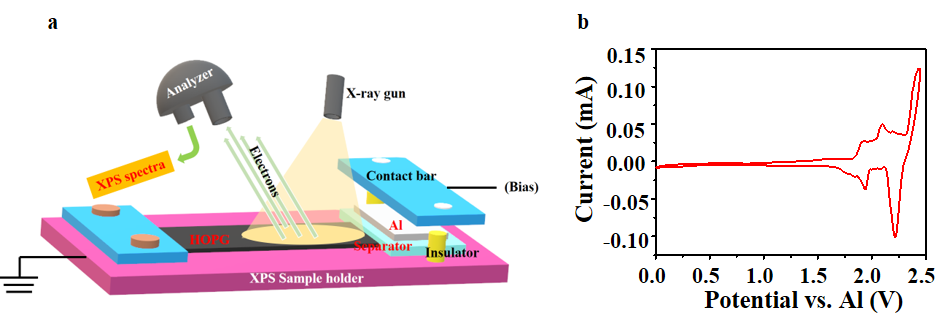


**Figure S1.** Sandwich Al | IL | HOPG model battery for operando XPS measurements. (a) The configuration of sandwich-like Al | IL | HOPG model battery for operando XPS measurements. The battery is mounted onto an Omicron-type direct-current heating sample holder. HOPG flake cathode is always ground during charging processes and XPS measurements, while the bias is applied on the contact bar which is in contact with the Al anode. In this model device, a small part of the graphene or graphite flake is in contact with the IL electrolyte while the major part of the graphene or graphite electrode surfaces are accessible for surface characterizations, e.g. XPS measurements. (b) CV data (0 - 2.45 V, 0.5 mV/s) acquired from the sandwich-like model battery mounted onto the XPS sample holder. The (de)intercalation redox peaks observed in the model batteries used in XPS measurements are similar with those observed in the planar model batteries and the real batteries [1].


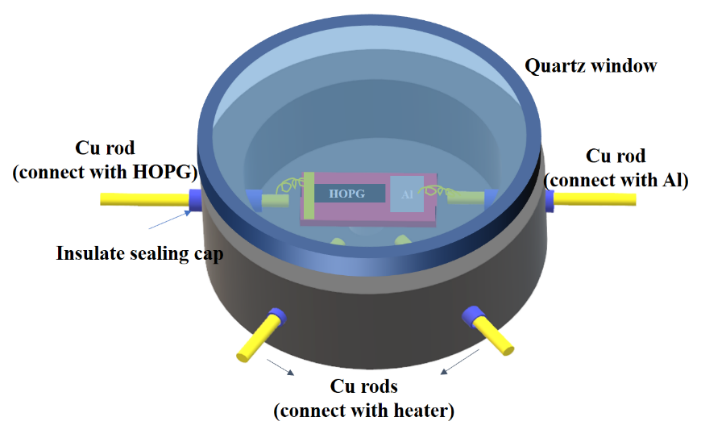


**Figure S2.** Optical cell for operando OM/Raman measurements. Planar model batteries were placed into the in-situ Raman cell equipped with a quartz window for operando Raman and OM measurements. The in-situ Raman cell has a CF35 flange with a quartz window and is sealed by the polytetrafluoroethylene gasket. Two copper wires are used to connect the work electrode (WE) and counter electrode (CE) contact pins.


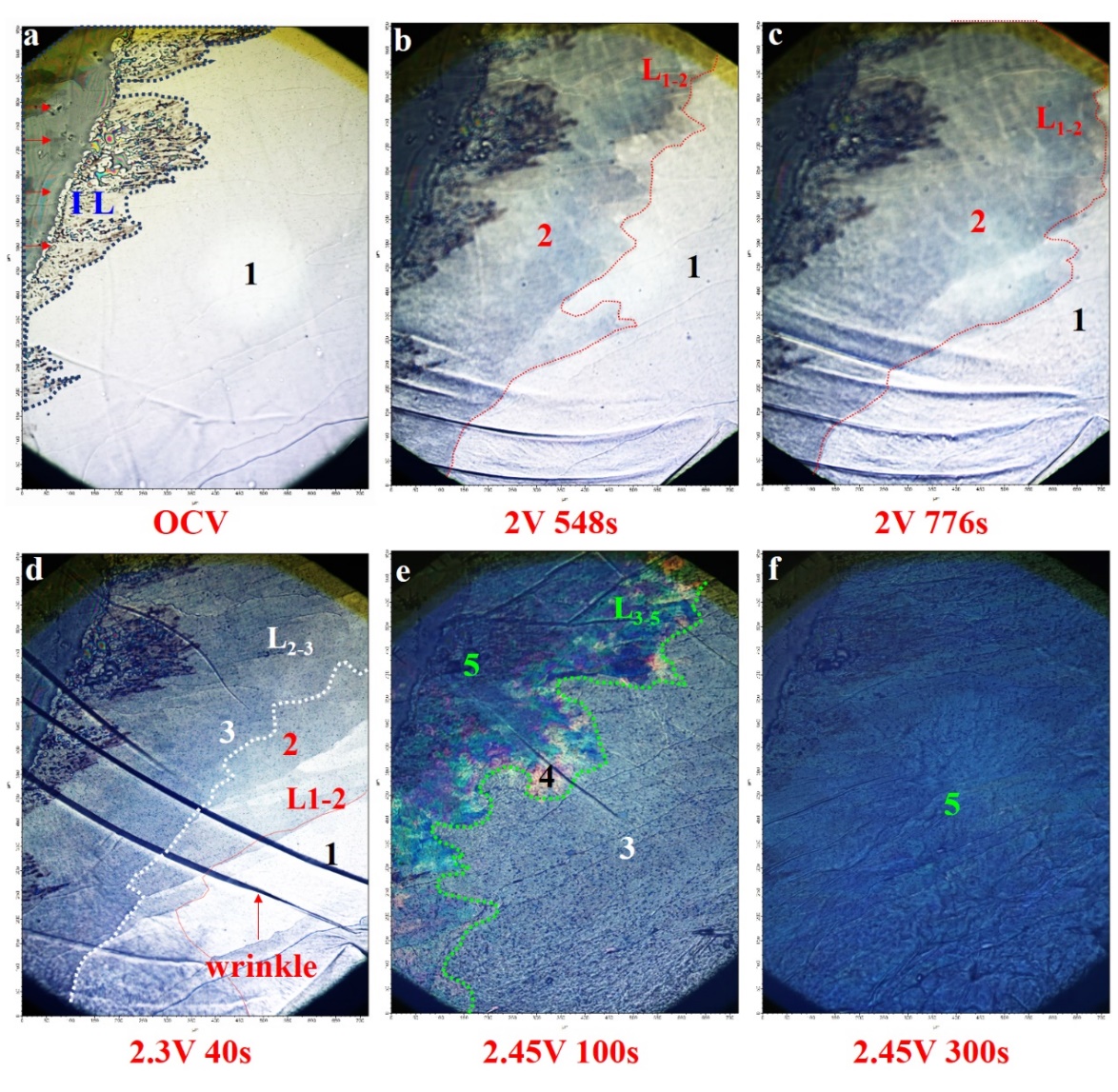


**Figure S3.** Operando optical microscope (OM) measurements. Another set of optical microscope images (700 × 950 μm^2^) captured during charging at different potentials and charging times (from supplementary movie 1-3). The distinct phase boundaries (dash lines) can be concluded by the different image contrast: 1. pristine; 2. gray; 3. light blue; 4. iridescence; 5. dark blue. The contrast is caused by different electronic structures of the graphite surface under the various intercalation states. In addition to the different contrast, morphology changes such as wrinkles and volume expansion can also be observed. Operando OM measurements elucidate that ions can intercalate into the graphene layers from the liquid/graphite interface and diffuse over a distance of millimeters away from the original interface. The intercalated ions form a uniform state at the graphite surface as shown by the uniform OM contrast. The OM results lay the foundation for the following operando XPS measurements since the X-ray spot size in our lab is around 5 mm. It should be mentioned that the observed OM contrast is not caused by electromigration of the electrolyte on the electrode surface since the IL drop edge has not been changed. The different optical contrast is caused by the modulated optical properties of the HOPG electrode during the intercalation [3, 4].

**Supplemental video captions**

**Video S1**

Real-time operando OM video imaging the lateral diffusion of the ions within HOPG flake in planar Al| IL| HOPG model battery at 2 V. The capture rate of the images is 0.5 Hz (2 s/ image) and the frame rate is 7 fps. The intercalated region can be distinguished by the distinct optical contrast (gray region illustrated in **Fig. S3a-c**) and the movement of diffusion frontiers can be clearly observed. In addition, the defocus of the images captured afterward is due to the volume expansion of HOPG flake during the intercalation.

**Video S2**

Real-time operando OM video imaging the lateral diffusion of the ions within HOPG flake in planar Al| IL| HOPG model battery at 2.3 V. The capture rate of the images is 0.5 Hz (2 s/ image) and the frame rate is 7 fps. A newly appeared contrast (light blue illustrated in **Fig. S3d**) was discovered and some dramatic morphology changes such as wrinkles can be observed.

**Video S3**

Real-time operando OM video imaging the lateral diffusion of the ions within HOPG flake in planar Al| IL| HOPG model battery at 2.45V. The capture rate of the images is 0.5 Hz (2 s/ image) and the frame rate is 7 fps. A new dark blue contrast and iridescence in boundary (illustrated in **Fig. S3e and f**) appeared.


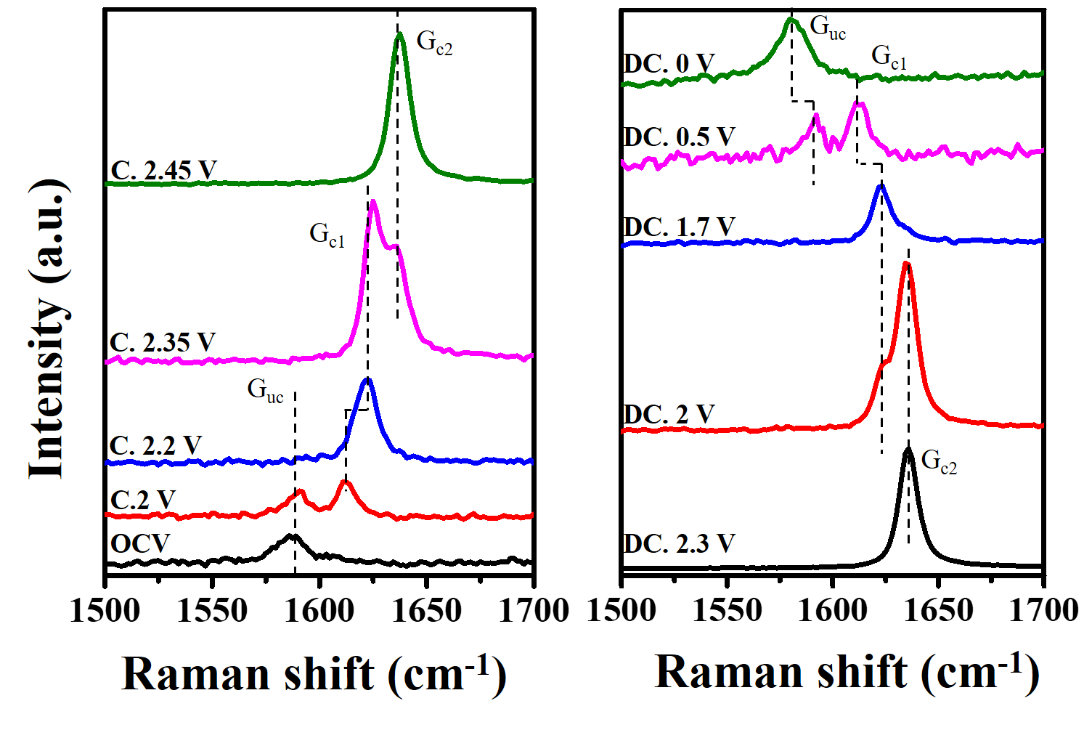


**Figure S4.** Selected operando Raman spectra of the HOPG electrode during charging (left) and discharging (right) processes. For the intercalation process (charging process), the evolution of G band of the HOPG electrode can be described as: G_uc_ → G_uc_ + G_c1_ → G_c1_ → G_c1_ + G_c2_ → G_c2_ (left). Upon the discharging, the evolution of G band is reversible, i.e. G_c2_ → G_c1_+G_c2_ → G_c1_ → G_uc_ + G_c1_ → G_uc_ (right). The Raman data confirm that the charging and discharging processes are reversible. The Raman measurement was carried out at the spot close to the electrolyte/graphite interface.

**Figure S5.** Operando XPS Al 2p measurements on the model AIB during the charging process**.** Each spectrum was recorded under the indicated charging condition as marked by the charging potential and charging time. The first spectrum was taken from the model electrode under the open circuit voltage (OCV) condition. The Al 2p signal in the first spectrum is from the surface contaminated electrolyte and its Al 2p binding energy is located at 75.7 eV. This binding energy is close to that of IL supported on a solid surface (Table S1)[5]. Upon charging, a new component at ~ 74.0 eV appears and its intensity keeps on increasing. It becomes saturated when charging at 2.45 V for 120 minutes. We attributed the newly appearing Al 2p signals to the intercalated Al species. Apparently, there is 1.7 eV binding energy difference between the surface adsorbed Al-containing species and the intercalated Al-containing species.

**Figure S6.** Operando XPS Cl 2p measurements on the model AIB during the charging process**.** Each spectrum was recorded under the indicated charging condition as marked by the charging potential and charging time. The first spectrum was taken from the model electrode under the OCV condition. The Cl 2p signal in the first spectrum is from the surface contaminated electrolyte and its Cl 2p binding energy is located at 199.6 and 201.3 eV from Cl 2p_3/2_ and Cl 2p_1/2_, respectively. Upon charging, new components at 197.9 and 199.6 eV appear and intensities keep on increasing. The newly appearing Cl 2p signals are attributed to the intercalated Cl species. Apparently, there is 1.7 eV binding energy difference between the surface adsorbed Cl-containing species and intercalated Cl-containing species.

**Figure S7.** Operando XPS N 1s measurements on the model AIB during the charging process. Each spectrum was recorded under the indicated charging condition as marked by the charging potential and charging time. The first spectrum was taken from the model electrode under OCV condition. The N 1s signal in the spectrum is from the surface contaminated electrolyte and its N 1s binding energy is located at 401.9 eV. Upon charging, a new component at 400.2 eV appears and its intensity keeps on increasing. The newly appearing N 1s signals are attributed to the intercalated N species (EMI^+^). Apparently, there is 1.7 eV binding energy difference between the surface adsorbed N-containing species and intercalated N-containing species (EMI^+^).


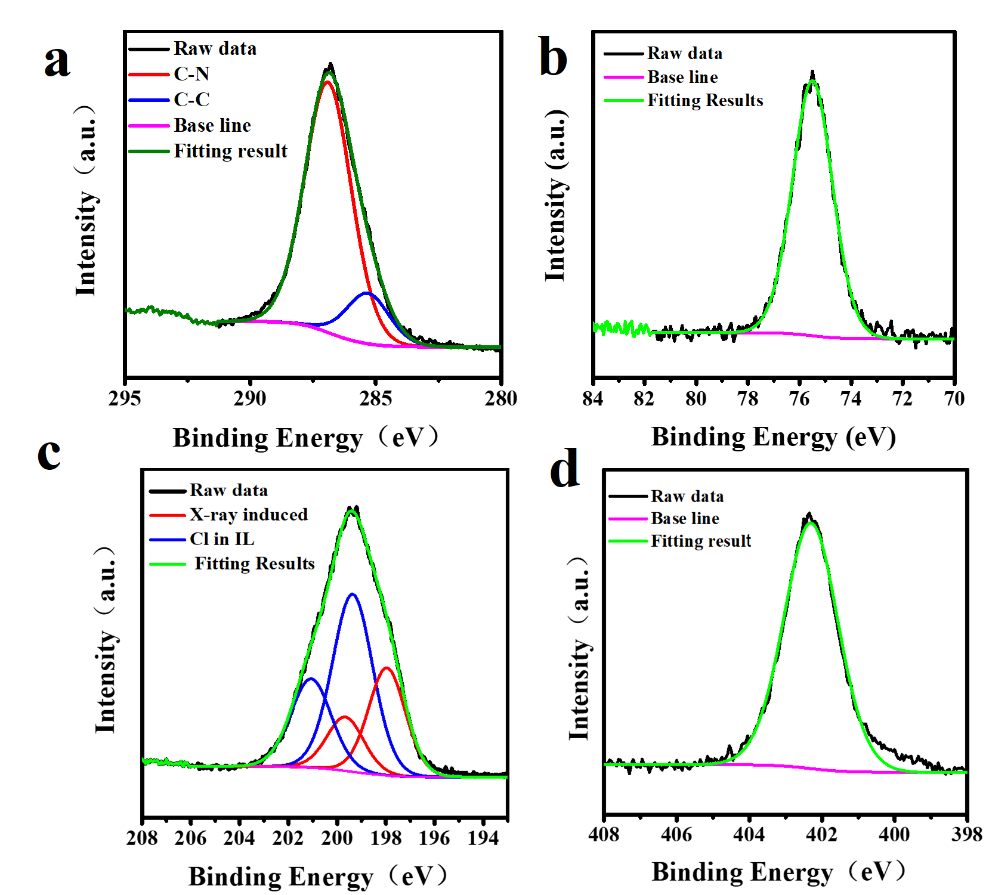


**Figure S8. Quasi in-situ XPS measurements of EMImCl/AlCl_3_ (1:1.3 by mole ratio) IL electrolyte supported on a Ta surface.** (**a**) XPS C 1s in C-N at 286.8 eV and C-C at 285.3 eV; (**b**) Cl 2p at 199.4 eV and 201.1 eV from Cl 2p_3/2_ and Cl 2p_1/2_, respectively. The unexpected Cl components labeled by red line are from [decomposition](javascript:;) [product](javascript:;) of IL induced by X-ray illumination; (**c**) Al 2p at 75.5 eV; (**d**) N 1s at 402.3 eV. The binding energy values are similar to the previous reports about XPS studies in supported ILs [5-10].

**Table S1. Binding Energies of Al 2p, Cl 2p_2/3_ and N 1s peaks from EMImCl/AlCl_3_ (1:1.3 by mole ratio) IL electrolyte supported on Ta foil, the IL adsorbed on HOPG electrode under the OCV condition, and fully charged HOPG electrode.** It can be concluded that the BEs of intercalated species are about ~1.7 eV lower than those of IL supported on solid surfaces.

| B.E. (eV) | Al 2p | Cl 2p_3/2_ | N 1s |
| --- | --- | --- | --- |
| IL on Ta | 75.5 | 199.4 | 402.3 |
| IL on HOPG at OCV | 75.7 | 199.6 | 401.9 |
| Fully charged HOPG | 74.0 | 197.9 | 400.2 |

**Figure S9. Operando XPS C 1s measurements on the model AIB during the charging process.** Each spectrum was recorded under the indicated charging condition as marked by the charging potential and charging time. The first spectrum was taken from the model electrode under OCV condition. The C 1s signal in the first spectrum with the main peak at 284.5 eV is mainly dominated by graphite surface C atoms. Upon charging, the C 1s intensity has been strongly weakened and its full width at half maximum (FWHM) keeps on increasing. During the charging, the remarkable volume expansion and surface roughness increase cause the dilution of surface C atoms in the XPS analysis regions. Furthermore, the increasing FWHM indicates that the chemical component of surface C atoms becomes more complicated after the intercalation. The newly appearing C atoms from intercalated EMI^+^ ions contain C-N at 285.1 eV/(C-N)_int_ and C-C at 283.6 eV/(C-C)_int_. The charged graphite C atoms have BE around 283.9 eV. The more detail is discussed in fig. S12.


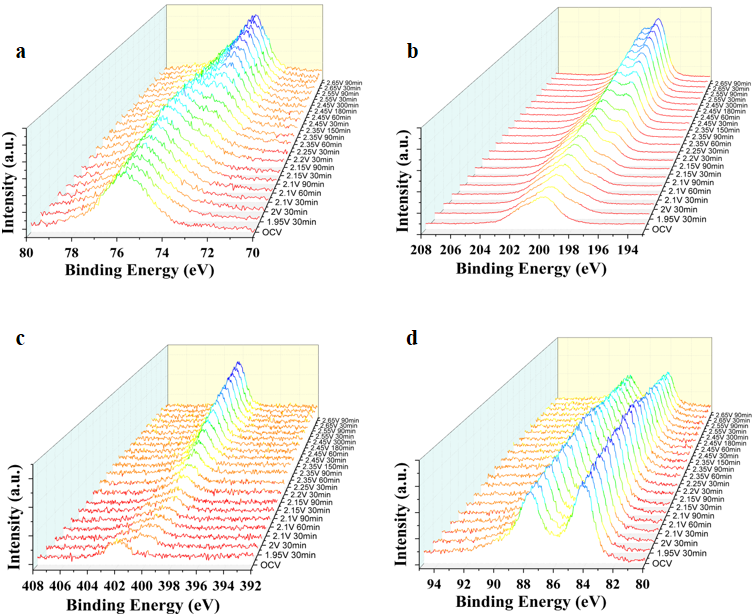


**Figure S10.** Calibration of XPS measurements over charging HOPG electrodes via dedicated deposition of Au overlayers. XPS Al 2p (a), Cl 2p (b), N 1s (c), and Au 4f (d) spectra during charging from OCV to 2.45 V. The intercalated Al, Cl, and N species all present around -1.7 eV binding energy shifts compared to the adsorbed species on the electrode surface. In contrast, the Au 4f spectra always present the same peak positions. The slight decrease in the Au 4f intensity is due to the increasing surface roughness. This experiment confirms that XPS measurements on the working electrodes are not influenced by the applied potentials.


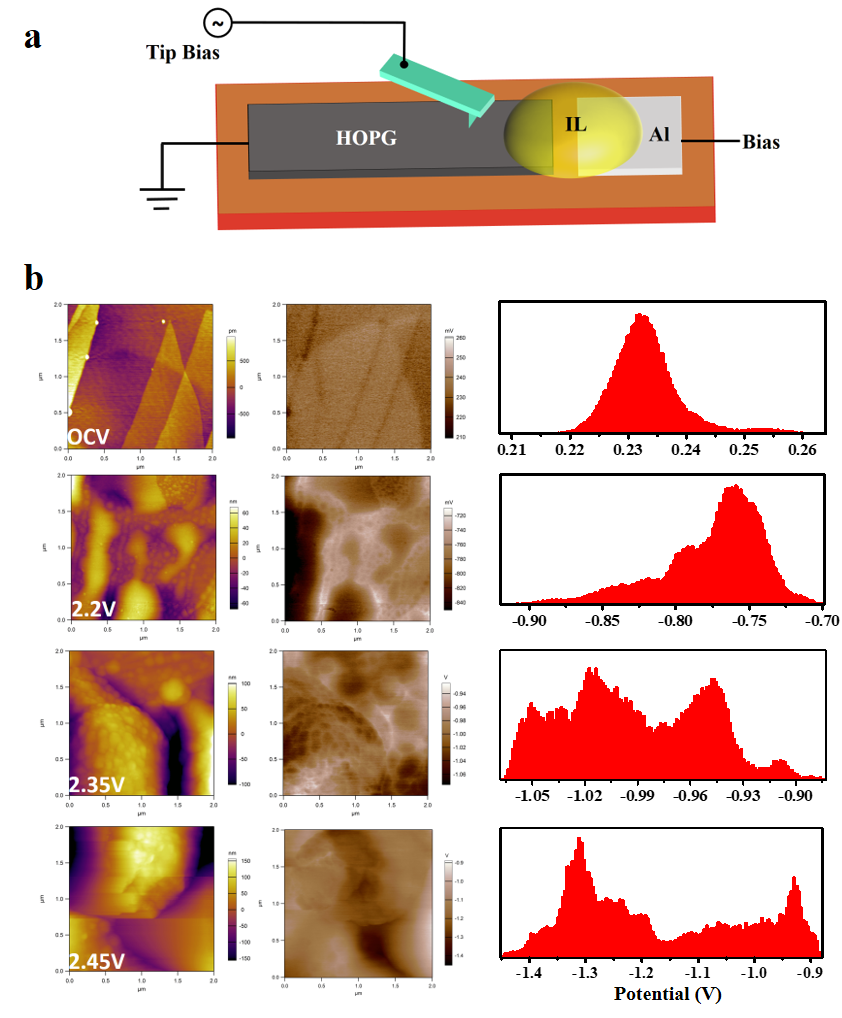


**Figure S11.** Operando SKPM measurements on the planar Al | IL | HOPG model battery**.** (a) Schematic for the operando SKPM measurements over the planar model battery. HOPG flake was ground and the charging potentials were applied to the Al foil. The AC voltage was applied at the AFM tip for surface potential measurements. **(**b) Selected height images (violet orange yellow), potential images (mud), and corresponding potential distribution results acquired at the charging potentials. The distribution of the surface potential in the right panel is collected from the total pixels in the potential image (mud, 256 × 256). The surface potentials exhibited in the figures and histograms are the values relative to the tip (φ_tip_ - φ_sample_)[11]. The surface roughness and surface work function were simultaneously increasing with the increasing charging potentials. At 2.45 V, the maximum decrease in the surface potential is close to 1.7 eV. It should be noted the SKPM measured the work function of host graphite surface rather than the guest intercalated ions measured by operando XPS measurements. The work function of the host graphite is gradually changed upon intercalation which is agree with our operando OM measurements.


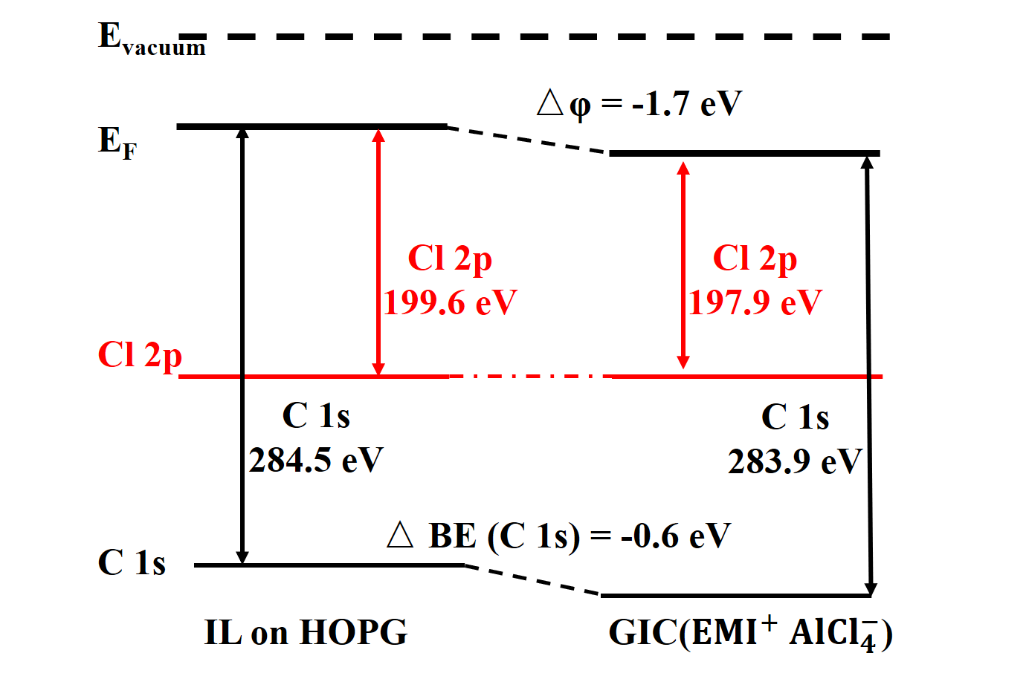


**Figure S12.** Energy schemes of IL supported on HOPG electrode and IL intercalated in HOPG electrode. Fermi level of the pristine HOPG electrode with surface adsorbed IL (left) is ~1.7 eV higher than that of the HOPG electrode with IL ions intercalated (right) as confirmed by the above SKPM measurement. Accordingly, the BE shifts of Al 2p, Cl 2p, N 1s, and C 1s spectra between surface adsorbed IL species and intercalated IL species can be attributed to the change in the surface work function induced by the charging process. C 1s spectra contain minor contribution from C atoms in the intercalated EMI^+^ ions having the similar 1.7 eV BE shift and major contribution from C atoms in the graphite, which is affected by both the change of the surface work function and their interaction with intercalated charges (see details in the next figure).


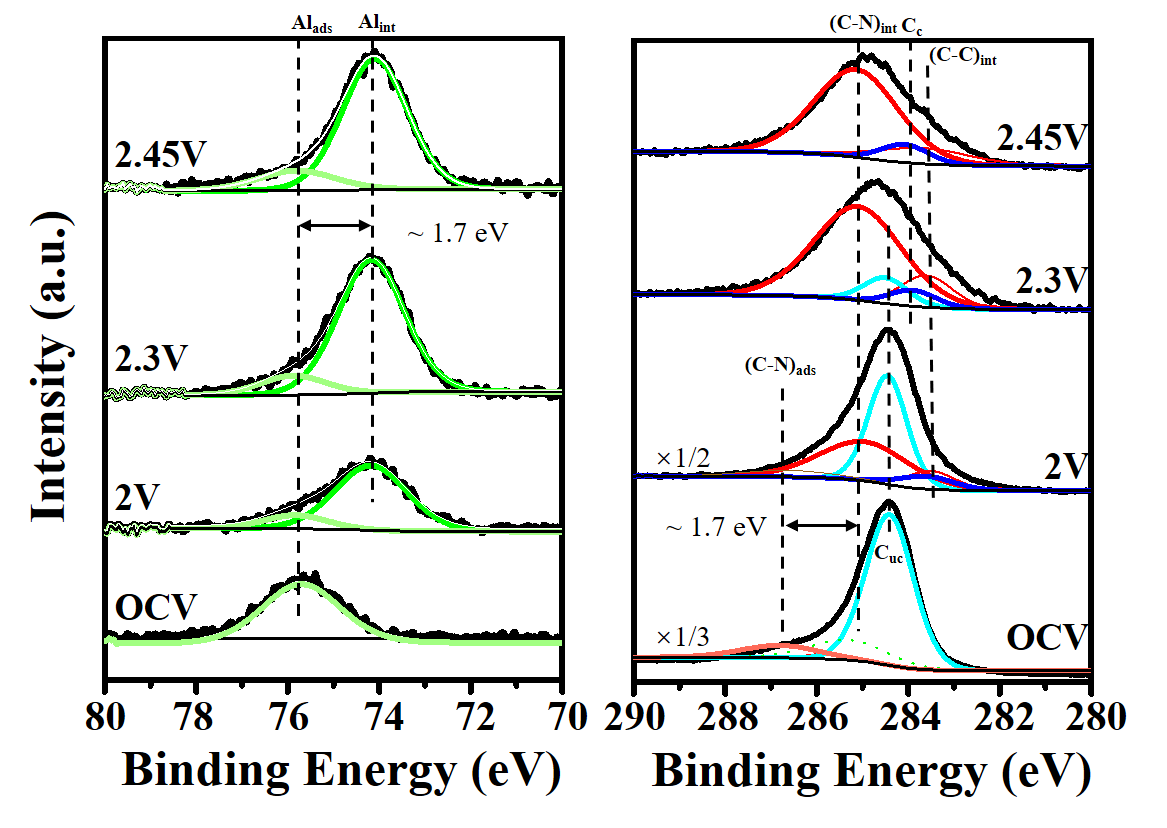


**Figure S13.** Operando XPS Al 2p (left) and C 1s (right) measurements on the model AIB battery during the charging process. C 1s spectra contain contributions from guest C atoms in intercalated EMI^+^ ions (C-N at 285.2 eV/(C-N)_int_ and C-C at 283.6 eV/(C-C)_int_) and host C atoms in the graphite electrode (uncharged graphite at 284.5 eV/C_uc_ and charged graphite at 283.9 eV/C_c_) regardless of some negligible C-O function group (green dash line).

C 1s components from guest C atoms in the intercalated EMI ions e.g. (C-N)_int_ should present the similar -1.7 eV BE shift compared with those of surface adsorbed EMI ions e.g. (C-N)_ads_. Meanwhile, C 1s components from host C atoms in the graphite electrode should be affected by both the change of the surface work function and their interaction with intercalated charges. C 1s component from the surface graphite C atoms without interaction with intercalants is labeled as C_uc_ having the peak position at 284.5 eV, while that of the surface graphite C atoms with interaction with intercalants is labeled as C_c_ having the peak position at 283.9 eV. The charge transfer from graphite C atoms to the intercalated ions results in the positive BE shift about +1.1 eV in the C 1s, while the change of the surface work function causes a BE shift of -1.7 eV. Overall, we see the BE shift of -0.6 eV for the graphene C atoms interacted with the charged ions, as shown in fig. S12.


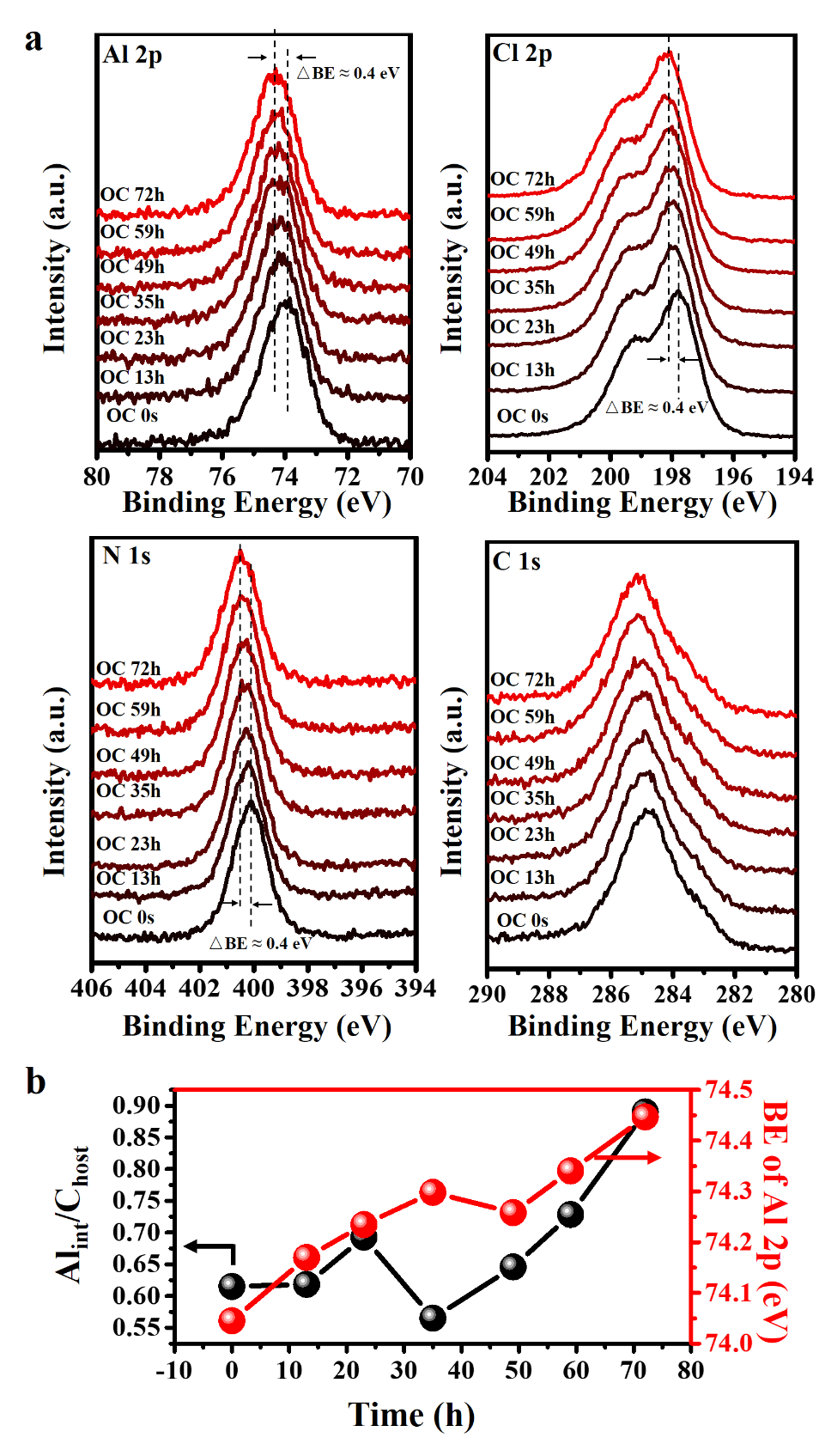


**Figure S14.** Operando XPS measurements under the OC condition. (a) XPS Al 2p, Cl 2p, N 1s, and C 1s spectra (normalized intensity) from the fully charged HOPG electrode surface when keeping OC for different times. All the spectra present +0.4 eV BE shifts after keeping OC for 72 h. (b) Atomic ratios between intercalated Al (Al_int_) and host C (C_host_) at different time (black sphere) and the BE of Al 2p at different OC times. During OC process, the Al_int_/C_host_ ratio increases slightly. If we consider the redistribution of intercalated anions and cations like the process in a supercapacitor under OC condition [12], the increase of intercalated Al species is expected. The results indicate that the relaxation and potential drop under OC condition is not caused by the de-intercalation of the ions but the redistribution of the ions and then change of the surface electronic state.


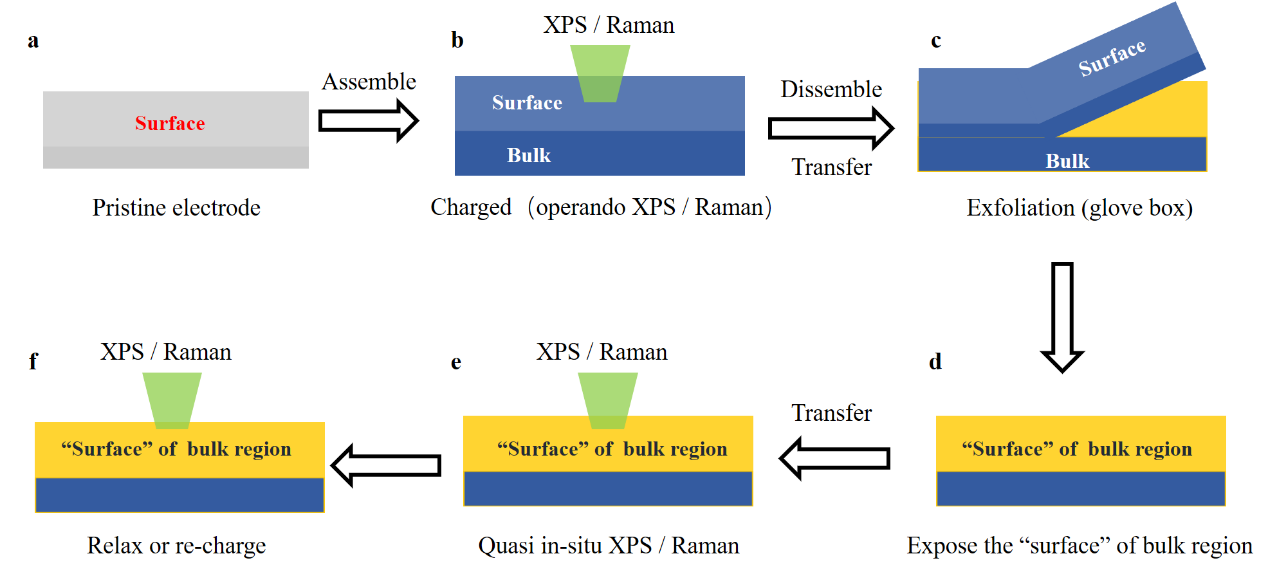


**Scheme S1.** Surface measurements on bulk regions of fully charged graphite electrodes.


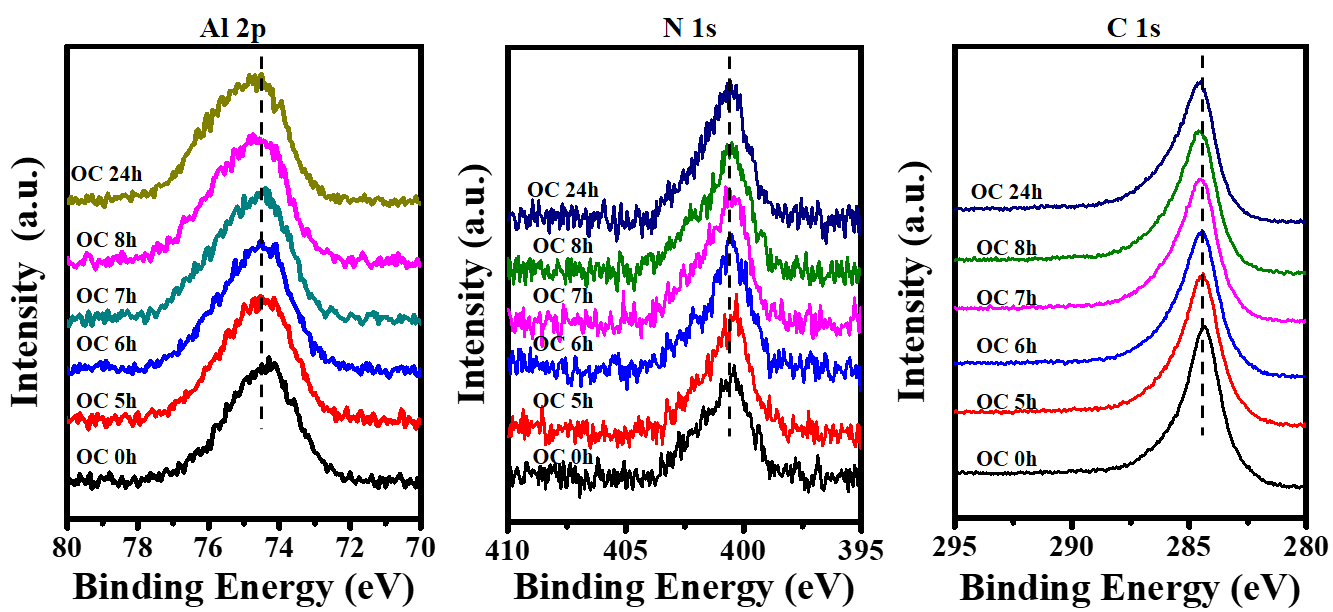


**Figure S15.** In-situ XPS measurements of exfoliated fully charged HOPG electrode under OC condition. Al 2p (left), N 1s (middle), and C 1s (right) spectra. No obvious changes were observed during OC state for various times.


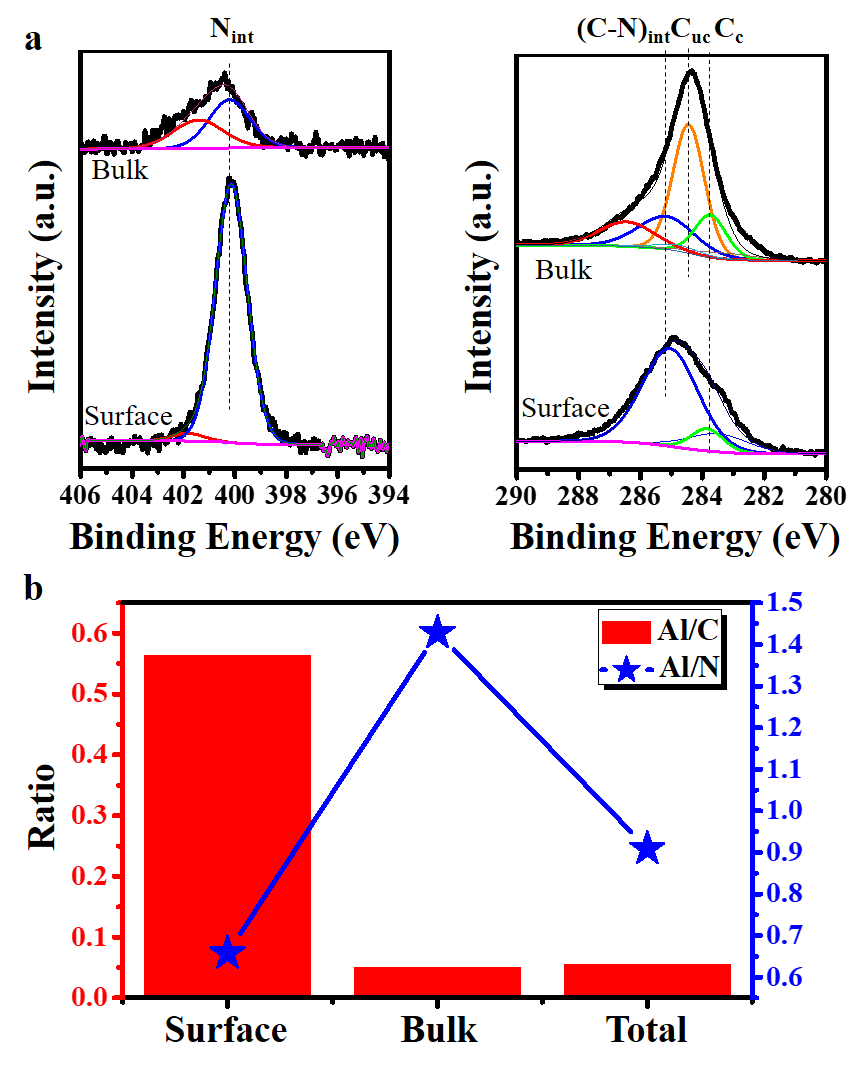


**Figure S16.** Comparative in-situ XPS measurements from surface region and bulk region of a fully charged HOPG electrode. (a), XPS N 1s (left) and C 1s (right) spectra acquired from as-charged graphite surface and bulk region of the electrode after exfoliation of the surface layer, respectively. Same as the Al 2p spectra (Fig. 3b), the signals from the bulk region have the similar BE positions as those from the surface but their intensities are all strongly weakened. In C 1s spectra from the bulk, C 1s components from host graphite C (both charged C atoms (C_c_) and uncharged C atoms (C_uc_)) become much stronger, while contributions from guest C in EMI ions, e.g. (C-N)_int_ are much smaller. (b) Al_int_/C_host_ and Al_int_/N_int_ ratios measured from the electrode surface and from the electrode bulk region based on XPS results. In addition, the chemical component of the total electrode was measured by chemical analysis methods including ICP, C/S element analyzer, and O/N/H element analyzer. The detail of the analysis process is presented in Materials and methods.


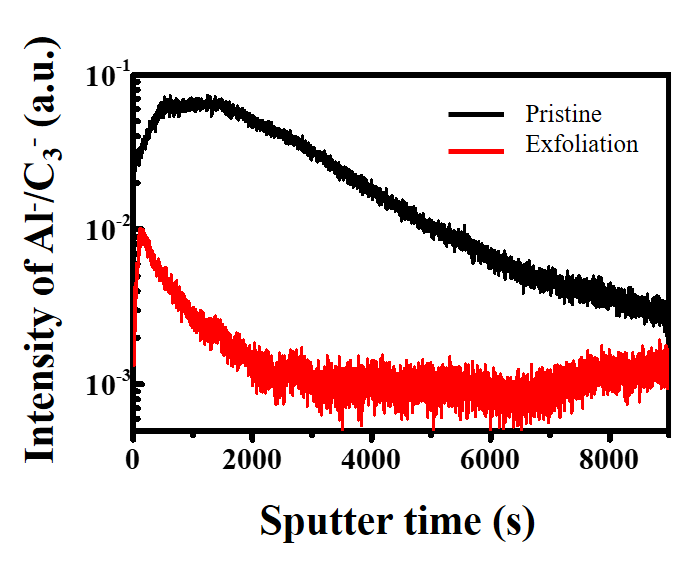


**Figure S17.** TOF-SIMS analysis of the fully charged HOPG electrode and the electrode subjected to surface exfoliation. The ratios between the Al^-^ and C_3_^-^ species as a function of the sputtering time from a pristine fully charged HOPG flake (black) and the exfoliated HOPG electrode (red) are shown. There is one-order higher intercalated concentration in the surface region compared to the bulk region. The sputtering energy and current of Cs are 2 KeV and 39.5 nA, respectively. The data points are realistic after sputtering for hundreds of seconds. It should be noted that the concentration gradient is present in the whole electrode from the top surface to the bulk based on the continuous decrease of the MS signals both in the pristine and exfoliated samples.


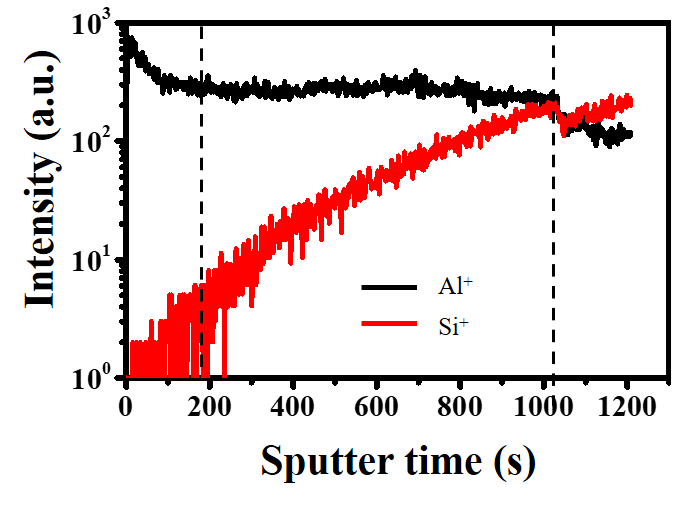


**Figure S18.** TOF-SIMS analysis of a fully charged ~500 nm thick graphene electrode on Si substrate. In order to [estimate](javascript:;) the thickness of the unusual surface region, ex-situ TOF-SIMS measurements were measured on a fully charged ~500 nm graphene electrode which was transferred and supported on a Si substrate. The sputtering energy and current of O are 2 KeV and 250 nA, respectively. The thickness of unusual region can be [estimate](javascript:;)d to be about less than 100 nm.


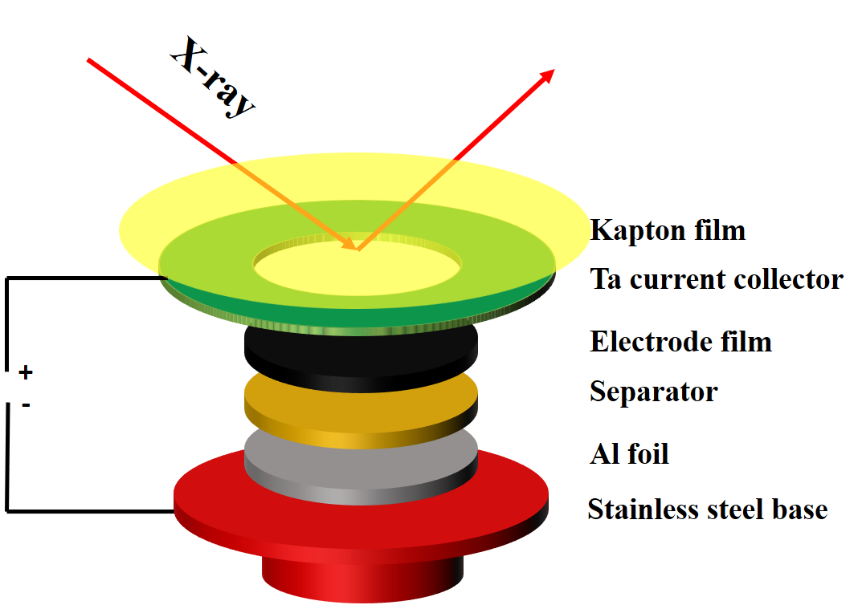


**Figure S19.** Schematic for operando XRD measurements. Sandwich AIB model battery was placed in an in-situ XRD cell. Graphite or graphene film was connected with a Ta current collector (with a hole) and covered by Kapton film. XRD data were recorded under the constant charging voltage (shown in Figs. 3c and 3d).


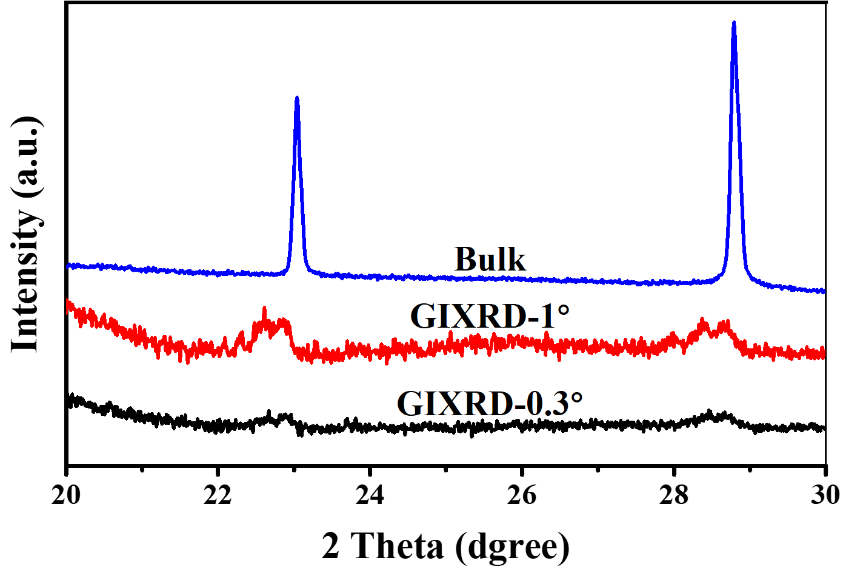


**Figure S20**. Ex-situ grazing incidence X-ray diffraction (GIXRD) of the fully charged HOPG electrode (covered by Kapton film). The degree marked on the curves represents the angle between the incidence X-Ray and the sample surface. The results are consistent with that obtained on the nanometer thick graphite film electrode suggesting the less ordered surface structure after the intercalation.


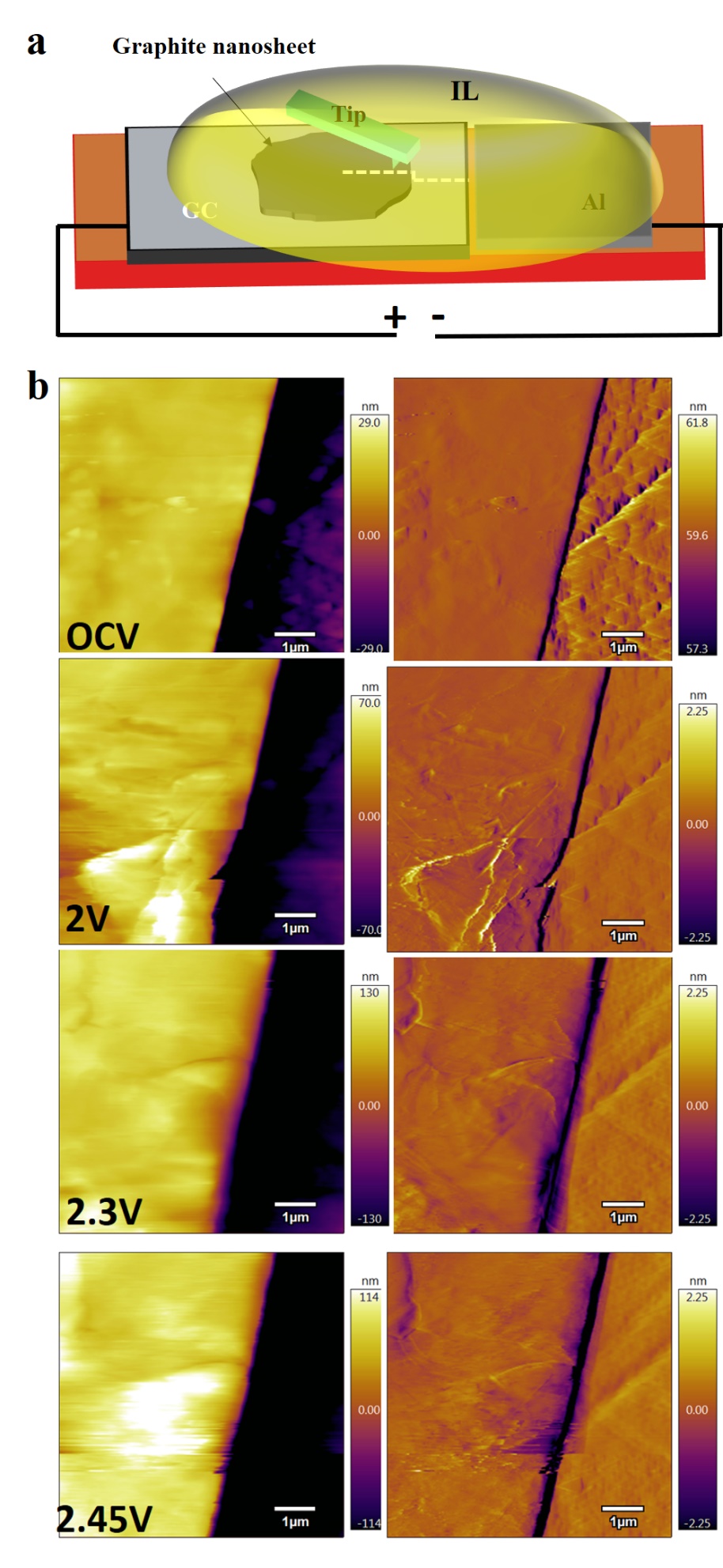


**Figure S21.** In-situ AFM measurements over a working graphite nanosheet. (a) In-situ AFM measurements (AC mode) were carried out over a mechanical exfoliated graphene nanosheet transferred onto a flat GC substrate. The thickness of the nanosheets is around some tens of nanometers. AFM tip (AC160TSA-R3-10, 250 Hz, 20 N/m) is totally immerged in the electrolyte drop and can measure the nanosheet heights during the charging. The set point amplitude of the tip is about 50% of the free amplitude. (b) Selected height (left) and amplitude (right) images from the in-situ AFM measurements on an exfoliated graphene nanosheet at each applied potential (applied for 20 min).


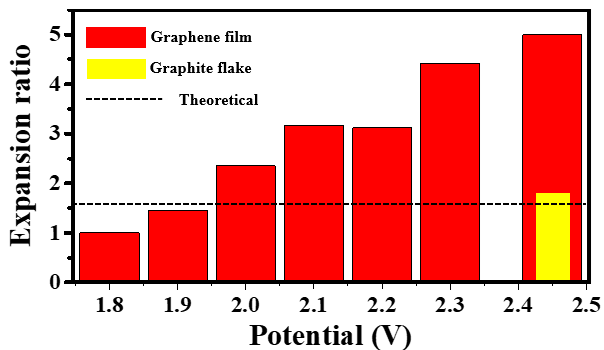


**Figure S22.** Expansion ratios calculated from the above in-situ AFM results. Theoretical reference value (~1.6 fold, the dashed line) is calculated based on a stage-3 GIC in ALB[13]. The measured expansion ratio of a micrometer thick graphite flake electrode is also included, which is based on the SEM measurements.


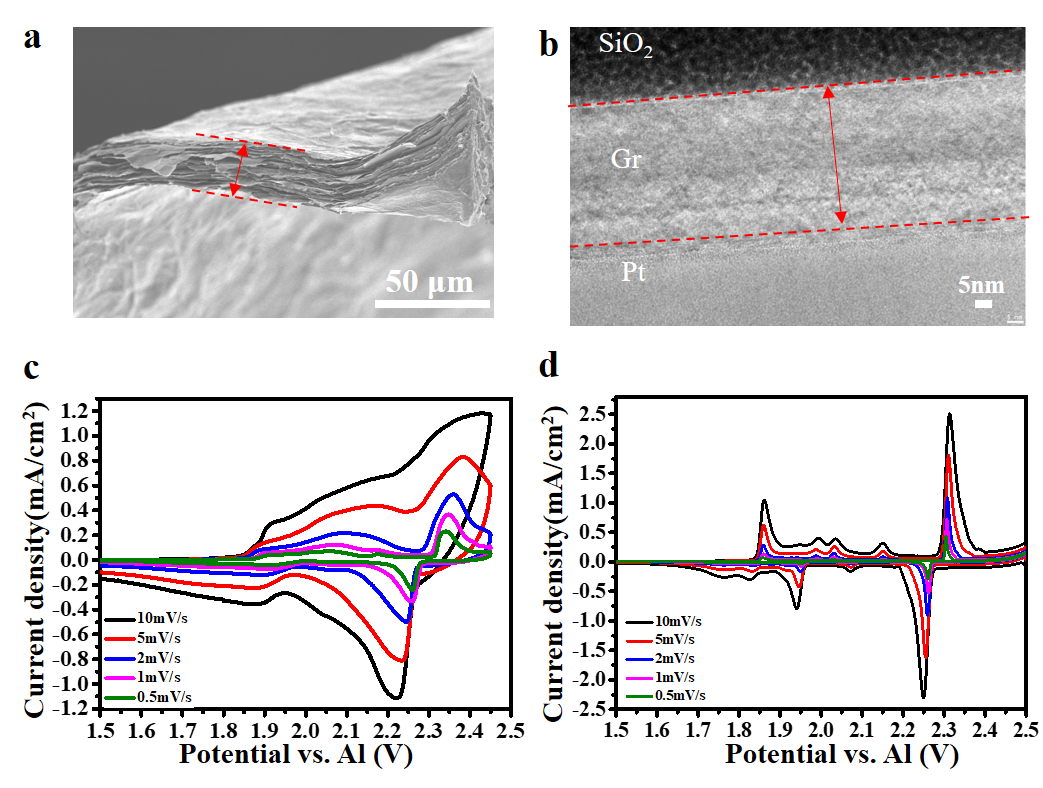


**Figure S23.** Structure and EC behaviors from thick graphite film and thin graphene film electrodes. (a) SEM cross section image of the thick graphite film. (b) TEM cross section image of the thin graphene film. These two films are also used in operando XRD measurements shown in Figs. 3c-3d. (c) CV curves from the model battery based on the thick graphite film electrode acquired at different scan rates. (d) CV curves from the model battery based on the thin graphene film electrode acquired at different scan rates. The EC measurements were performed at the three-electrode mode.


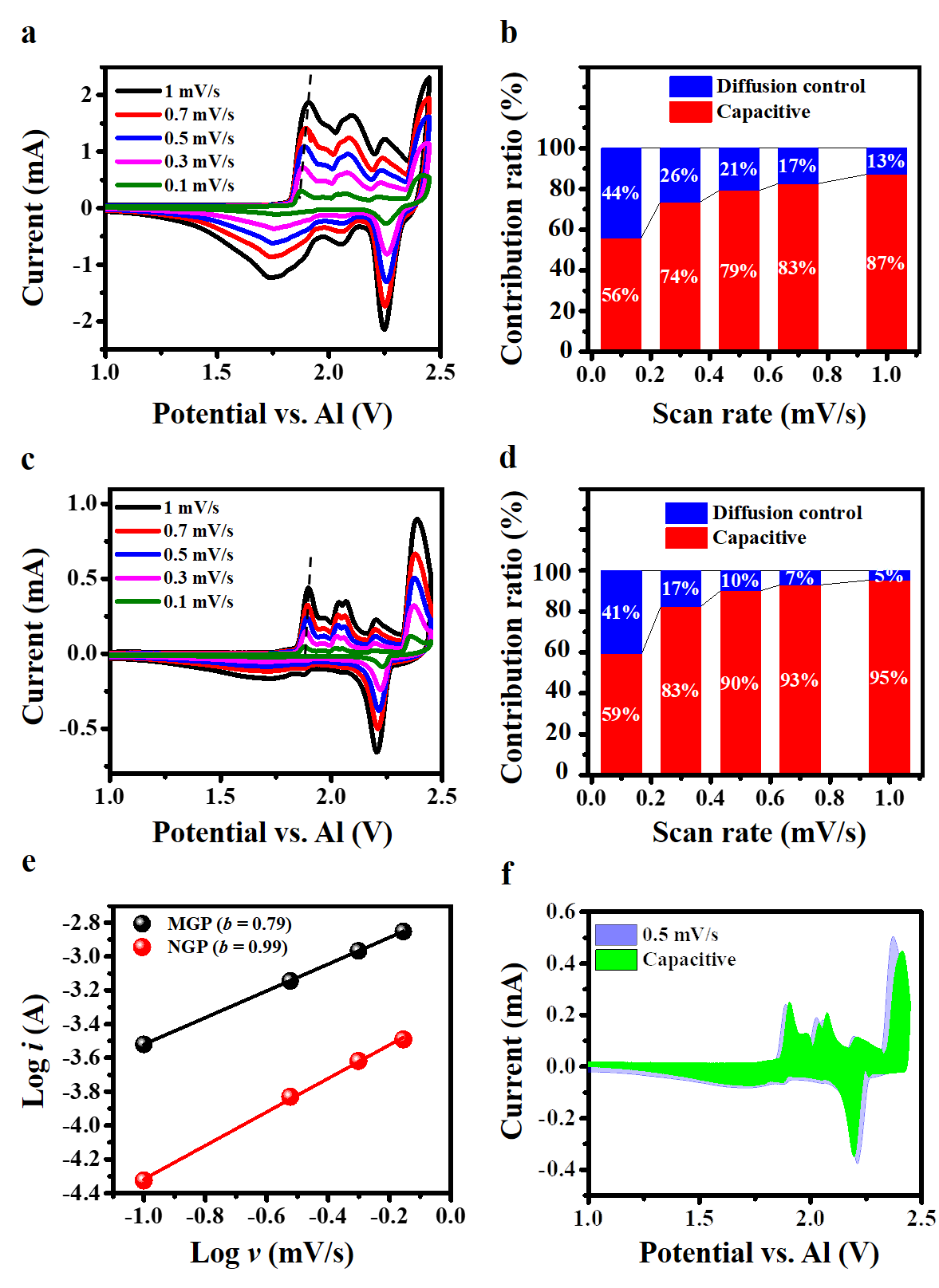


**Figure S24.** EC behaviors of two real coin-type batteries using MGP and NGP cathode (described in Fig. 4c). (a) and (c), CV curves from the batteries using MGP (a) and NGP (c) as the cathode materials at different scan rates (1 - 0.1 mV/s). The 1^st^ intercalation peak (marked by dash line) currents (*i*) vs. scan rates (*v*) were fitted according to the relation[14] of*i = av^b^*. The *b* value derived from the MGP based device is 0.79, while the *b* value from the NGP based device is 0.99 indicating the intercalation pseudo capacitance charging mechanism in the device (e). Accordingly, the contributions from capacitance in the MGP-based device (b) and in the NGP-based device (d) are illustrated by calculating *k_1_*(capacitive controlled process) and *k_2_* (diffusion-controlled process) [15, 16] in *i*(V) *= k_1_v + k_2_v^1/2^*. The capacitive distribution in the NGP-based device at 0.5 mV/s was shown in (f) as an example. The capacitive controlled process is dominant in the NGP-based device.


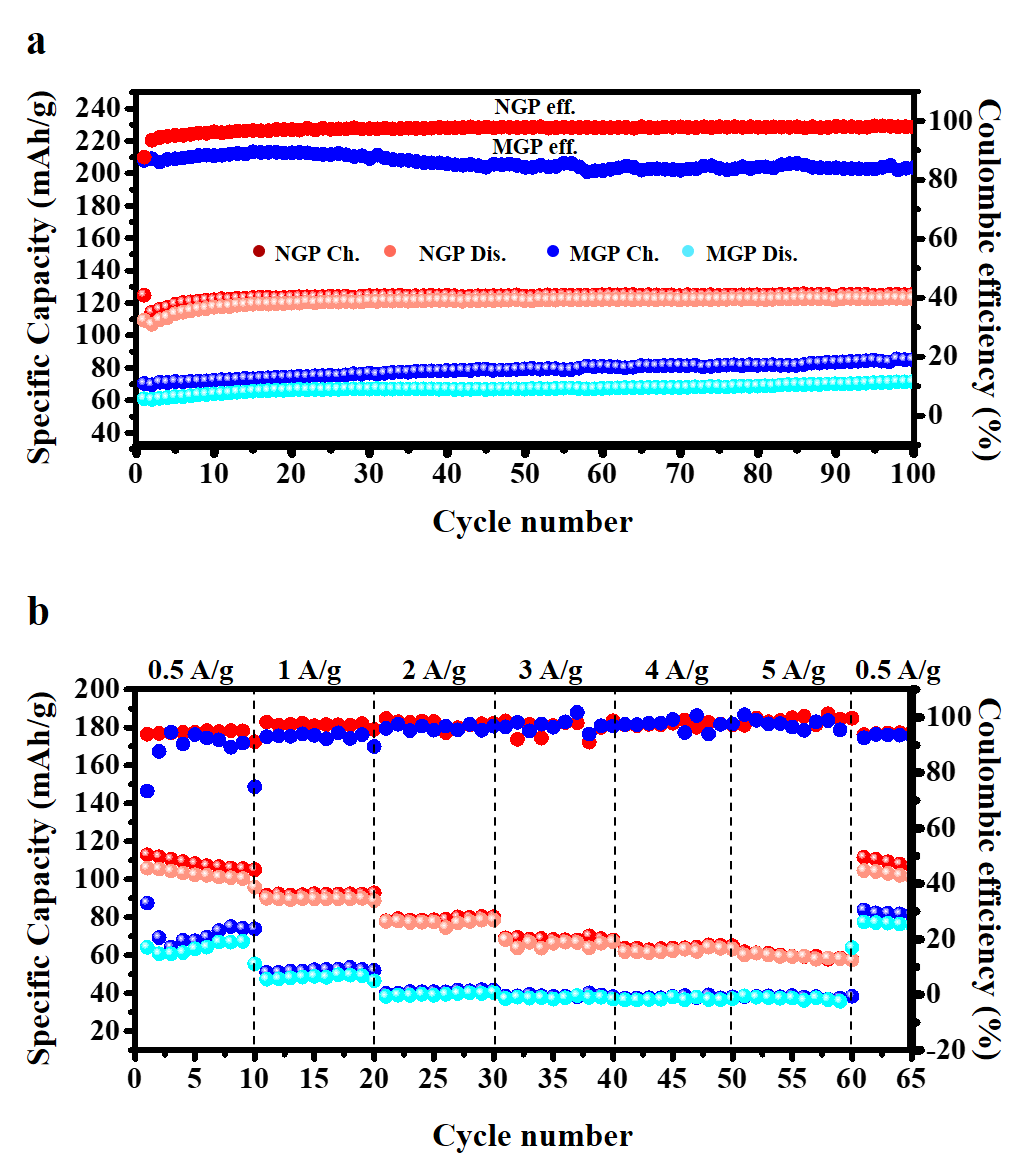


**Figure S25.** Electrochemical performance of two real coin-type cells using NGP and MGP as the cathode material, respectively. (a) Long-term cycling stability of MGP-based and NGP-based cathodes tested at 0.5 A/g. The NGP-based cathode shows better performance with the specific capacity about one-fold higher than that of the MGP-based cathode and presents high coulombic efficiency in 100 cycles. (b) Rate capability of MGP-based and NGP-based cathodes at different current densities. It should be mentioned that the better rate capability of the NGP-based cathode is another critical factor of the corresponding higher capacity.


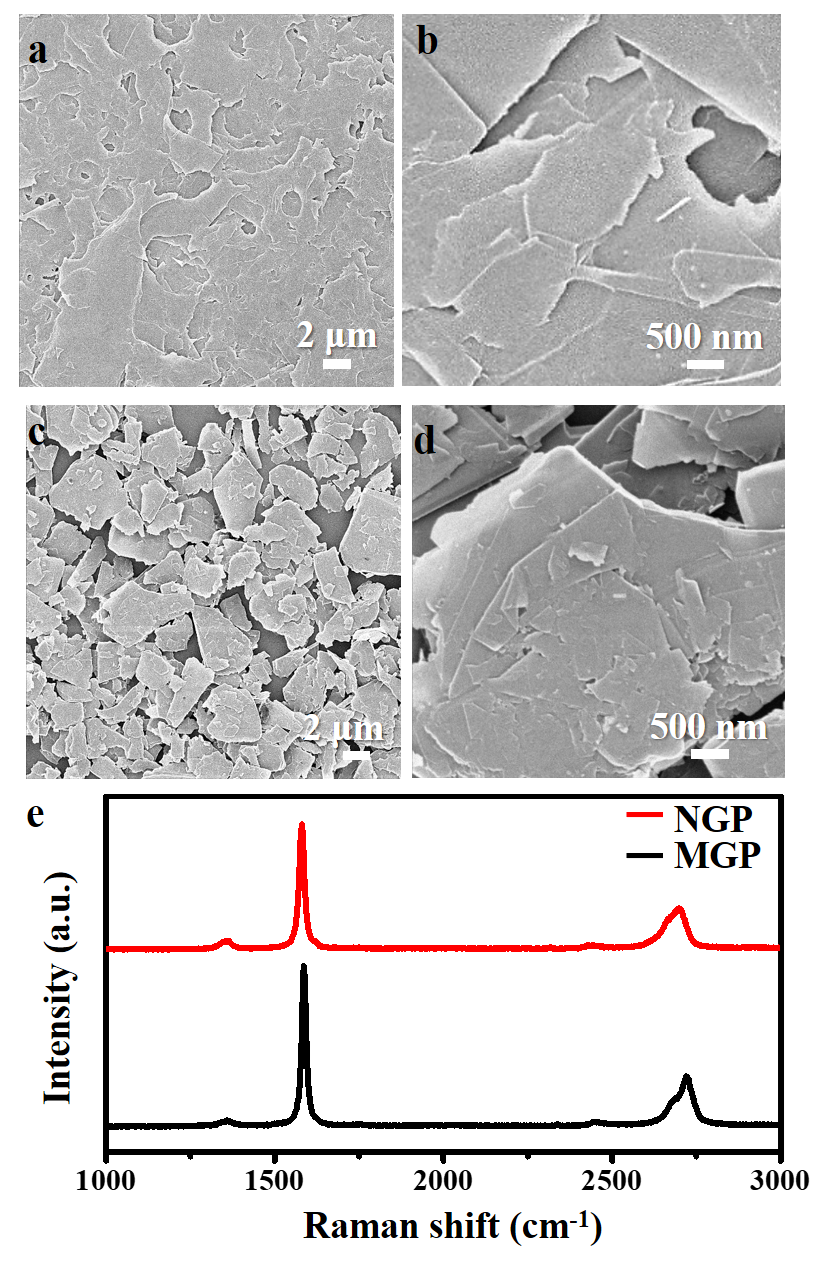


**Figure S26.** Structure characterization of MGP and NGP cathodes. (a) and (b), SEM images of the NGP cathode. (c) and (d), SEM images of the MGP cathode. (e) Raman spectra of the NGP and MGP materials. It should be noted that the lateral size and the concentration of defect of both two materials are similar. Consequently, the difference in the electrochemical performance is solely due to the thickness effect or the surface effect as revealed by our operando surface science measurements.

**REFERENCE**

1. Lin MC, Gong M and Lu BG*, et al.* An ultrafast rechargeable aluminium-ion battery. *Nature* 2015; **520**: 325-8.

2. Angell M, Pan CJ and Rong YM*, et al.* High coulombic efficiency aluminum-ion battery using an AlCl_3_-urea ionic liquid analog electrolyte. *Proc Natl Acad Sci U S A* 2017; **114**: 834-9.

3. Bao W, Wan J and Han X*, et al.* Approaching the limits of transparency and conductivity in graphitic materials through lithium intercalation. *Nat Commun* 2014; **5**: 5224.

4. Dimiev AM, Ceriotti G and Behabtu N*, et al.* Direct real-time monitoring of stage transitions in graphite intercalation compounds. *ACS Nano* 2013; **7**: 2773-80.

5. Moulder JF, Stickle W and Sobol P*, et al.* Handbook of X-ray photoelectron spectroscop. *Physical electronics, Minnesota* 1992: 44-5.

6. Rietzler F, Nagengast J and Steinrück HP*, et al.* Interface of ionic liquids and carbon: ultrathin [C_1_C_1_Im][Tf_2_N] films on graphite and graphene. *J Phys Chem C* 2015; **119**: 28068-76.

7. Steinruck HP. Recent developments in the study of ionic liquid interfaces using X-ray photoelectron spectroscopy and potential future directions. *Phys Chem Chem Phys* 2012; **14**: 5010-29.

8. Steinrück H-P and Wasserscheid P. Ionic Liquids in Catalysis. *Catal Lett* 2014; **145**: 380-97.

9. Villar-Garcia IJ, Smith EF and Taylor AW*, et al.* Charging of ionic liquid surfaces under X-ray irradiation: the measurement of absolute binding energies by XPS. *Phys Chem Chem Phys* 2011; **13**: 2797-808.

10. Lovelock KR, Villar-Garcia IJ and Maier F*, et al.* Photoelectron spectroscopy of ionic liquid-based interfaces. *Chem Rev* 2010; **110**: 5158-190.

11. Nonnenmacher M, Oboyle MP and Wickramasinghe HK. Kelvin probe force microscopy. *Appl Phys Lett* 1991; **58**: 2921-3.

12. Kaus M, Kowal J and Sauer DU. Modelling the effects of charge redistribution during self-discharge of supercapacitors. *Electrochim Acta* 2010; **55**: 7516-23.

13. Pan CJ, Yuan C and Zhu G*, et al.* An operando X-ray diffraction study of chloroaluminate anion-graphite intercalation in aluminum batteries. *Proc Natl Acad Sci U S A* 2018; **115**: 5670-5.

14. Arico A, Bruce P and Scrosati B*, et al.* Nanostructured materials for advanced energy conversion and storage devices. *Nat Mater* 2005; **4**: 366-77.

15. Sathiya M, Prakash AS and Ramesha K*, et al.* V_2_O_5_-anchored carbon nanotubes for enhanced electrochemical energy storage. *J Am Chem Soc* 2011; **133**: 16291- 9

16. Bard A. Electrochemical methods: fundamentals and applications. 2001.
